# Towards Exploring Toxin-Antitoxin Systems in *Geobacillus*: A Screen for Type II Toxin-Antitoxin System Families in a Thermophilic Genus

**DOI:** 10.3390/ijms20235869

**Published:** 2019-11-22

**Authors:** Rawana N. Alkhalili, Joel Wallenius, Björn Canbäck

**Affiliations:** 1 Biotechnology, Department of Chemistry, Lund University, SE-221 00 Lund, Sweden; 2 Department of Biology, Lund University, SE-221 00 Lund, Sweden; joelwallenius@gmail.com (J.W.); Bjorn.Canback@biol.lu.se (B.C.)

**Keywords:** AbrB-ParE, c-di-AMP, *Geobacillus kaustophilus*, *Geobacillus thermodenitrificans*, *Geobacillus thermoleovorans*, *Geobacillus* sp. ZGt-1, GNAT-HTH, (p)ppGpp, MNT-HEPN, XRE-COG2856

## Abstract

The toxin-antitoxin (TA) systems have been attracting attention due to their role in regulating stress responses in prokaryotes and their biotechnological potential. Much recognition has been given to type II TA system of mesophiles, while thermophiles have received merely limited attention. Here, we are presenting the putative type II TA families encoded on the genomes of four *Geobacillus* strains. We employed the TA finder tool to mine for TA-coding genes and manually curated the results using protein domain analysis tools. We also used the NCBI BLAST, Operon Mapper, ProOpDB, and sequence alignment tools to reveal the geobacilli TA features. We identified 28 putative TA pairs, distributed over eight TA families. Among the identified TAs, 15 represent putative novel toxins and antitoxins, belonging to the MazEF, MNT-HEPN, ParDE, RelBE, and XRE-COG2856 TA families. We also identified a potentially new TA composite, AbrB-ParE. Furthermore, we are suggesting the *Geobacillus* acetyltransferase TA (GacTA) family, which potentially represents one of the unique TA families with a reverse gene order. Moreover, we are proposing a hypothesis on the *xre-cog2856* gene expression regulation, which seems to involve the c-di-AMP. This study aims for highlighting the significance of studying TAs in *Geobacillus* and facilitating future experimental research.

## 1. Introduction

The toxin-antitoxin (TA) system-related research is an evolving field. Most of the TA modules in bacteria were discovered during the first decade of the current millennium, and studies have indicated that TA genes are encoded on plasmids and/or chromosomes of almost all bacteria and many archaea [1,2]. The significance of the TA system lies in regulating cell growth and death to help prokaryotes cope with different stress conditions [2].

The TA system is composed of a stable toxin protein and a cognate labile antitoxin that is either a protein or an RNA neutralizing the toxin [3]. There are six types of TA systems that differ in terms of the antitoxin nature and the mechanism of neutralizing the toxin [3]. Type I and II are the most abundant in prokaryotes. Type II has been widely studied [1], but it has not been well-studied in thermophilic bacteria, including *Geobacillus* (see below).

In the type II TA system, the antitoxin is a protein that forms a stable complex with the toxin to neutralize it and block its toxic activity under normal growth conditions [2,4]. Under stress conditions, the antitoxin is degraded by a protease, releasing the stable toxin to interact with its cellular target and influence a cellular process that could have a bacteriostatic or a bactericidal effect [1,5]. Even members of the same toxin protein superfamily differ significantly in their amino acid (aa) sequences, and thus have different interactions with different cellular targets [6]. TA-coding genes are often found in an operon and are thus co-regulated [7]; however, this is not necessarily the case all the time, as explained in the Results and Discussion section. TA-coding genes may overlap [8], and the antitoxin-coding gene is often located upstream of the toxin-coding gene. This order guarantees synthesis of the antitoxin before that of the toxin [2]. However, exceptions have been reported [2].

TAs can be present as one pair or more per cell [5]. The plasmid-encoded TA loci are often associated with plasmid stabilization via a mechanism known as post-segregational killing (PSK) [1]. While there is evidence that some chromosomally encoded TA pairs are involved in genome stability via the PSK mechanism as well [1,3,5], such as the case with the RelBE TA family of *E. coli*-K12 [9] and ParDE in *Vibrio* spp. [10,11], the roles for many are varied and still debated [8]. Chromosomally encoded TA pairs may be involved in responses of bacterial cells to various stress conditions [1,3,8], including nutrient deficiency and bacteriophage infection, as shown for the *E. coli* MazEF TA family [12,13], and exposure to antibiotics and formation of persisters, as shown for various type II TA families of the pathogenic *Salmonella* spp. [14]. They may also be involved in bacterial programmed cell death (PCD), which can be described as an altruistic suicide, a role that was described for the MazEF TA family in different *E. coli* strains [15,16] but has been opposed [17], and may as well be involved in biofilm formation [1,5], such as the case with the MqsRA TA family in different *E. coli* strains [18]. Furthermore, TAs may help bacteria in colonizing niches [3,5], such as the case with the PasTI TA family of the pathogenic *E. coli* ExPEC strain [19], and play a role in virulence in pathogenic bacteria [5,8], such as the case with the PezAT TA family of *Streptococcus pneumoniae* [20] and SezAT TA family of *Streptococcus suis* [21].

Regulation of the TA modules is connected to cellular signaling pathways [3]. Cell signaling molecules, such as the (p)ppGpp (guanosine tetra or pentaphosphate, known as the alarmones [22], play a vital role in the regulation of the transcription and translation of TA modules, and thus bacterial physiology [3]. The (p)ppGpp is a global regulatory signal in bacteria, induced by nutrient-starvation and, in turn, activates the toxin by activating proteases that degrade the antitoxin [3,23] (the (p)ppGpp role is discussed further in Section 2.8.1). The opposite seems to also be possible for some toxins depending on their mechanism of action, as Álamo et al. have shown that in *Bacillus subtilis*, the type II toxin ξ, an ATPase, hydrolyzed the ATP, converted the GTP to (p)ppGpp, and raised the levels of the second messenger, the cyclic-di-adenosine monophosphate (c-di-AMP) [24]. Quorum-sensing signal molecules may also be involved in activating the type II toxins, as discussed further below.

Studies of TA systems have focused on mesophiles, while thermophilic bacteria have not been given the same attention. Apart from what has been reported in the TA database (TADB) [25], there is only one study that was conducted on a thermophilic bacterial strain, *Thermus thermophilus*, to analyze the VapBC TA module [26]. Given the stress conditions that thermophilic bacteria encounter in their ecological niches, studying the roles and mechanisms of their TA systems may broaden our knowledge on these entities that help bacteria cope with stress. It may also further elucidate the adaptation strategies employed by thermophilic bacteria. Moreover, studying the TA systems of thermophilic bacteria could shed some light on their potential biotechnological applications. Genome analysis aiming for mining genomes of thermophilic strains for TAs will facilitate the future experimental work, as it will help in defining the TAs of interest, and thus will set a path for experimental design.

Due to our interest in the identification of the antibacterial potential of *Geobacillus* spp. in general, and *Geobacillus* sp. strain ZGt-1 in particular [27,28], we have mined the genomes of certain *Geobacillus* strains that we are interested in for type II TA system families, as a first step towards its experimental investigation. For the TA screening, we selected *Geobacillus* sp. strain ZGt-1, which was isolated from Zara hot spring in Jordan [27,29], and we also selected the type strains of species that showed the closest similarity to strain ZGt-1, as indicated in [27]. The strains we selected were isolated from different ecological systems; *G. kaustophilus* strain HTA426 was isolated from the deep-sea sediment of the Mariana Trench [30] and *G. thermoleovorans* strain CCB_US3_UF5 (hereafter referred to as “Gts” (for *G. thermoleovorans* strain), for simplicity) was isolated from a hot spring in Malaysia [31]. We selected *G. thermodenitrificans* NG80-2 as well, since it was isolated from an oil reservoir in China, which is a non-aquatic ecological niche [32]. The present study aims for the identification of TA-coding genes, excluding the ones residing within the genome region of the integrated prophage. 

**Figure 1:**
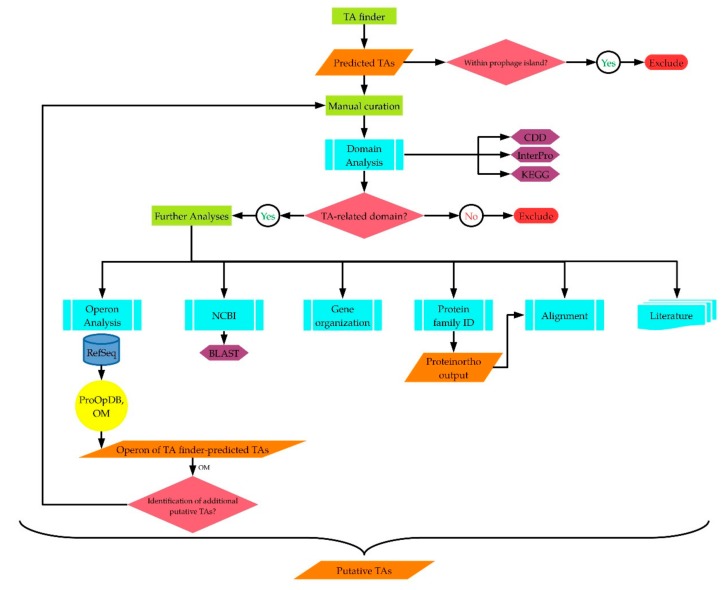
Workflow chart summarizing the analysis approach carried out in this study for the identification of type II TA families.

Studying the TA systems of phylogenetically related strains isolated from different ecological systems will give indications about the diversity of these systems. Furthermore, mining genome sequences for TA genes will reveal their presence, since a number of them have been overlooked during the genome annotation or annotated as coding for hypothetical proteins [33]. Various analysis tools were used (Figure 1) and resulted in the identification of 28 putative TA pairs on the chromosomal genomes of the strains. Among the identified TAs, 15 represent putatively novel ones. The term “novel” in this context refers to toxin and antitoxin proteins that have not been identified in the literature as putative toxins/antitoxins of the respective *Geobacillus* strain, and have either been overlooked or annotated as hypothetical in both genome records, the original and the RefSeq, of the analyzed *Geobacillus* type strains. For the non-type strain, *Geobacillus* sp. ZGt-1, the protein is described as novel if it has been annotated by the NCBI as hypothetical or if the NCBI blastp result showed a hypothetical protein as the top hit. The identified TAs are discussed below.

## 2. Results and Discussion

In this study, the strategic approach plotted for the in-silico TA identification involved several steps (Figure 1). The workflow started with predicting the TAs of the four *Geobacillus* strains using the TA finder software [25]. This was followed by inspecting the genome context of the putative TA-coding genes. TAs whose genes were located within prophage islands, as indicated from the neighboring genes, were excluded. The included putative TAs were then manually curated. The protein domains of each toxin and antitoxin were identified using the CDD (Conservation Domain Database) and InterPro prediction tools. The KEGG Genes database and the KEGG KOALA BLAST [34] were used in cases where the CDD and InterPro tools did not predict a protein domain, as explained in the text. At this stage, proteins that did not harbor toxin-/antitoxin-related domains were excluded. The proteins that harbored related domains were further analyzed using various tools. The NCBI blastp showed the annotation of the putative toxin/antitoxin. Those annotated as hypothetical proteins in the RefSeq and original genome records, or their genes had not been annotated have been described here as putative novel toxins/antitoxins, as mentioned above. The organization of the putative TA-coding genes on the genome was also inspected, to see if the putative antitoxin-coding gene was upstream of the toxin-coding gene, or if they had a reverse gene order. The Operon Mapper tool [35] was used for predicting whether the putative TA-coding genes were potentially sharing the same operon or not. The Prokaryotic Operon Database (ProOpDb) [36] was used when the Operon Mapper did not predict the operon for the type strains. Worth mentioning here is that the Operon Mapper helped in identifying additional TAs, since some TA-coding genes identified by the TA finder software were shown, using the Operon Mapper, to share the operon with a third gene. Protein domain analysis of the third gene sometimes showed a toxin-/antitoxin-related domain. Therefore, these proteins were subjected to the same manual curation steps mentioned above, as shown in Figure 1. The resulting TAs were aligned based on their protein families; i.e., their type II TA families (Figures S1–S10). In parallel to these analysis steps, we mined the literature for the features of the TA families of the identified putative TAs and checked whether these TAs had already been reported or not yet.

Employing this approach, we identified 28 putative TA pairs on the chromosome of each of the four *Geobacillus* strains analyzed here, whereas there were no type II TA genes detected on the plasmids of any of the strains. The predicted TA pairs are distributed over eight TA families (Table 1). Additionally, in three of the strains, we identified two and three apparently solo putative toxins and antitoxins, respectively, that could be acting together with the predicted TA pairs (Table 1). Out of the identified putative TAs, 15 represent putatively novel ones as they were not recognized previously as part of the TA system of the given *Geobacillus* strain (Table 2). Below, follows a description of the identified TA families.

### 2.1. GNAT-HTH (GacTA)

A TA pair composed of a toxin harboring the GNAT domain (GCN5-related N-acetyltransferases, originally derived from GCN5 (general control non-repressible 5), a histone acetyltransferase [37]), and an antitoxin harboring the HTH (Helix-Turn-Helix) domain or its variants, RHH (Ribbon-Helix-Helix) [38] or wHTH (winged Helix-Turn-Helix) belongs to type II TA families [25,39,40,41,42].

Each of the four *Geobacillus* strains harbors one GNAT-wHTH pair. We suggest calling this TA family “GacTA” (*Geobacillus* acetyltransferase toxin-antitoxin) in accordance with the previously reported RHH-GNAT TA families, KacAT of *Klebsiella pneumonia* [43] and TacAT of *Salmonella enterica* serovar Typhimurium [44], while also considering the reverse order of the TA-coding genes, as discussed below.

#### 2.1.1. GacTA of G. kaustophilus HTA426 and G. thermoleovorans Gts

The genes with the locus tags GK1498 and GTCCBUS3UF5_17280 (the locus tag prefix “GTCCBUS3UF5” will hereafter be referred to as “*”, for simplicity) in strains HTA426 and Gts, respectively, code for a putative GNAT domain-harboring toxin (Table 1).

There is a difference in the aa sequence of the putative encoded toxin between the original and RefSeq genome annotations. Based on the global alignment of the aa sequences of GNAT toxins (Figure S1a) of the four *Geobacillus* strains, it is more likely that the originally annotated putative toxin has the correct aa sequence. Therefore, we considered it for our analysis (Table S1). Nevertheless, the one annotated in the RefSeq record also harbors the GNAT domain. The aa sequences of the two putative toxins are identical between strains HTA426 and Gts (Table S1; global alignment in Figure S1a).

The adjacent gene, GK1499 in strain HTA426, and *_17290 in strain Gts, codes for a putative wHTH-harboring antitoxin (Table 1). 

The intergenic region between the putative genes of the TA pair of strain HTA426 is 128 nucleotide (nt) long, and that of strain Gts is 131 nt. Here as well, there is a difference in the aa sequence between the original and RefSeq genome annotations in strain HTA426, but it is only a slight difference where the antitoxin of the originally annotated genome has a duplicated start aa (methionine; M) (global alignment in Figure S1b). This slight difference did not affect the domain analysis. We considered the putative antitoxin annotated in the original genome record for our analysis (Table S1), since this was the record, we had to consider for the putative GNAT toxin, as discussed above. Apart from this extra M aa, the aa sequences of the two putative antitoxins are identical between the two strains (global alignment in Figure S1b).

#### 2.1.2. GacTA of *Geobacillus* sp. ZGt-1

Analysis of the genome sequence of strain ZGt-1 indicated that the gene on contig 16_17 codes for a putative GNAT domain-harboring toxin (Table 1). The putative wHTH-harboring antitoxin is encoded by the adjacent gene on contig 16_18 (Table 1). The intergenic region between the putative genes of the TA pair is 131 nt long. NCBI blastp supports our results (Table S2), and the aa sequences of this putative TA pair are almost identical to those of strains HTA426 and Gts (global alignment in Figure S1).

#### 2.1.3. GacTA of *G. thermodenitrificans* NG80-2

The gene with the locus tag GTNG_1349 codes for a putative GNAT domain-harboring toxin (Table 1). There is a slight difference in the aa sequence of the toxin between the original and RefSeq genome annotations. The RefSeq annotated toxin is better aligned with the GNAT toxins of the other strains (Figure S1a). Therefore, we considered it for our analysis (Table S1). Nevertheless, the putative toxin annotated in the original genome record also has a GNAT domain. The start codon of GTNG_1349 is GTG, instead of the common ATG.

The adjacent gene with the locus tag GTNG_1350, annotated in the original and RefSeq genome records, codes for a wHTH-harboring putative antitoxin (Table 1). The intergenic region between the putative genes of the TA pair is 127 nt long. The aa sequences of the GacTA pair of this strain showed a few aa differences compared to the pairs of the other three strains (Figure S1).

Furthermore, the TA finder identified GTNG_1577 and GTNG_1578 as a TA pair. However, our manual curation indicated that both genes with these locus tags code for putative GNAT toxins (Table 1 and Table S1) that did not align with the other GNAT toxins in the four strains (Figure S1). The putative genes coding for these two putative toxins are potentially regulated by the same operon (Table 1). In a trial to identify the antitoxin-coding gene(s), we analyzed the neighboring genome context. The analysis revealed the presence of a putative gene with the locus tag GTNG_1575, harboring an HTH domain (Table 1). This putative gene is encoded on the opposite DNA strand and is not sharing the operon with a toxin. It has been reported previously that the interaction within a TA pair operated by different operons, despite being uncommon, is possible [45,46,47]. Accordingly, it could be that those two GNAT-coding genes and the HTH-coding gene form a TA system. Although a TA system is usually a two-component system composed of a toxin and an antitoxin, cases of a three-component system have also been reported [48,49] and they seem to be distributed in bacteria more commonly than previously thought [50]. It is interesting to note that the previously reported three-component systems have one toxin each, while the GacTA system reported here has two adjacent putative toxins. Carrying out experimental work will clarify the actual components of this system. On the other hand, since GNATs can acetylate the amino group of a broad variety of substrates and thus are involved in various cellular processes [51], it could be that the identified GNAT-harboring proteins of the *Geobacillus* strains do not act as part of a TA system.

Regarding the GNAT-wHTH pairs of the four *Geobacillus* strains, our analysis showed that the putative toxin and its cognate antitoxin are potentially regulated by two separate, yet adjacent operons. We noticed that while the gene coding for the putative GNAT toxin is the only gene in its operon, the gene coding for the putative antitoxin is potentially regulated by the adjacent operon together with four to seven other genes. It could be that the operon prediction algorithm mistakenly separated the two adjacent genes coding for the putative TA pair into two operons. When we manually checked the distance between the genes of each TA pair, we found that it is within the range 128–132 nt, making it hard to deduce whether the toxin- and antitoxin-coding genes share the same operon or not. It could also be that the two putative TA genes are potentially regulated by two separate operons, as mentioned above. However, based on functional annotations of the other genes sharing the operon with the putative antitoxin-coding one, these genes are not related to TA-coding or regulating genes (Table S3). Therefore, it is more likely that the TA-coding genes were mistakenly predicted as regulated by two separate operons. It should be noted that in contrast to most TA systems, the order of the genes coding for the putative GNAT-wHTH TA pair in the four *Geobacillus* strains shows that the toxin-coding gene is upstream of the antitoxin-coding one. This atypical gene order has been reported only in few TA families; HigBA [52], MqsRA [53], and HicAB [54]. Regardless of which of the two mentioned alternatives is correct, the gene order of this GNAT-wHTH TA family highlights the significance of researching it in *Geobacillus*, as it represents one of the potentially unique TA families. Experimental analysis of the RNA expression and the possible post-transcriptional regulations will demonstrate the special protein expression mechanisms that these *Geobacillus* strains employ to secure the production of wHTH antitoxin to neutralize the GNAT toxin, as has already been done for *E. coli* strains that harbor HigBA, MqsRA, and HicAB (for details, the reader is referred to [54,55,56,57,58,59]. Worth mentioning is that the HTH (RHH)-GNAT TA family, which has been experimentally studied so far, was of the bacterial strains *K. pneumoniae* HS11286 [43] and *Salmonella enterica* subsp. *enterica* serovar Typhimurium LT2 [60], where the TA-coding genes have the typical gene order. Therefore, the *Geobacillus* strains are showing a previously unreported gene order of the putative GNAT-HTH TA family.

In summary, our analysis indicated that all the four *Geobacillus* strains have a putative GNAT-wHTH TA family, or GacTA, whose aa sequences are either identical or highly similar (global alignment in Figure S1). The putative wHTH-GNAT TA family represents a potentially unique TA family with atypical TA gene order. Therefore, experimental studies of the *Geobacillus* putative GNAT-wHTH TA family will be of significance.

### 2.2. MazEF

The MazEF (ma-ze means “what is it?” in Hebrew [61]) family is among the most well characterized TA families [2]. However, the role of the MazF toxin has been debated whether it is bacteriostatic [17,62,63] or bactericidal, where the cell reaches a “point of no return” and undergoes the PCD [15,64,65,66]. Kolodkin-Gal et al. demonstrated that the PCD is mediated by a quorum-sensing signal molecule, a pentapeptide known as the extracellular death factor (EDF) [67], representing another example on the integration of TA modules into the cellular signaling pathways mentioned above. The MazEF TA family includes different TA composites, since MazF may pair with antitoxins other than MazE, a phenomenon described as a “mix and match” between toxin and antitoxin superfamilies [68]. For instance, MazF may pair with antitoxins harboring the RHH or AbrB (AidB regulator domain) domains. The TA family of these different composites is also classified as MazEF [69] (for details, the reader is also referred to [2,22,70]). Bacterial species may have more than one pair of MazEF homologs [71]. This is the case with the *Geobacillus* strains analyzed in this study, as discussed below.

#### 2.2.1. MazEF (I) –MazE-MazF Composite

##### MazEF (I) of *G. kaustophilus* HTA426, *Geobacillus* sp. ZGt-1, and *G. thermoleovorans* Gts

The genes with the locus tags GK1648 and *_19090 in strains HTA426 and Gts, respectively, and the gene encoded on contig 16_162 in ZGt-1 code for a putative MazF domain-harboring toxin, here labeled as MazF (I) (Table 1). In the three strains, each of the genes with the locus tag GK1647 and *_19080, and that encoded on contig 16_161 code for a putative MazE antitoxin, here labeled as MazE (I) (Table 1). Each of these genes is adjacent to and upstream of its cognate toxin-coding gene. The two genes coding for each of the putative TA pairs potentially share the operon in each of the strains and overlap by one nt. The one nt overlap between TA pair genes is common among TA systems [8]. The aa sequences of the three putative toxins are identical among the three strains, and the same applies to the putative antitoxins (Table S1; global alignment Figure S2). In each of the three strains, the NCBI annotation of the putative TA pair supports our results (Table S1). The fourth strain, *G. thermodenitrificans* NG80-2 does not have the MazE-MazF composite.

#### 2.2.2. MazEF (II)–RHH-MazF Composite

##### MazEF (II) of *G. kaustophilus* HTA426, *Geobacillus* sp. ZGt-1, and *G. thermoleovorans* Gts

The genes with the locus tags GK0233 and *_2500 in strains HTA426 and Gts, respectively, code for a putative MazF domain-harboring toxin, MazF (II) (Table 1) belonging to the PemK toxin superfamily. The gene encoded on contig 4_61 in ZGt-1 also codes for MazF (II) (Table 1).

In strain Gts, there is a difference in the aa sequence of the putative toxin between the original and RefSeq genome annotations. Nevertheless, in both records, the protein harbors the same domain that identifies it as a putative MazF toxin. For our analysis, we considered the RefSeq annotation, since the MazF annotated in the RefSeq record is better aligned with the toxins of the other analyzed *Geobacillus* strains (Figure S3a). Each of the putative toxin-coding genes has been annotated as coding for a “type II toxin-antitoxin system endoribonuclease” in the genome records of the three strains (Table S1). Furthermore, our results specified the type of the endoribonuclease to be MazF.

The analysis also indicated that in the three strains, the genes with the locus tags GK0232 and *_2490, and the gene encoded on contig 4_60 code for an RHH-domain harboring antitoxin (Table 1). The antitoxin-coding gene is adjacent to and upstream of its cognate toxin-coding one in all three strains. The two genes coding for each of the putative TA pairs share the same putative operon in each of the strains (Table 1), and there is no overlap between the two genes of any TA pair. In each of the three strains, the putative antitoxin-coding gene has been annotated by NCBI as coding for a hypothetical protein (Table S1). However, using the results of our analysis, we could identify each of these three hypothetical proteins as a putative RHH domain-harboring antitoxin (Table 2). The aa sequences of the three putative toxins are identical among the three strains, and the same applies to the putative antitoxins (Table S1; global alignment Figure S3).

##### MazEF (II) of *G. thermodenitrificans* NG80-2

The gene with the locus tag GTNG_0207 codes for a putative MazF toxin (Table 1). The aa sequence of the putative toxin is identical to MazF toxins in the three other *Geobacillus* strains mentioned above (Table S1; global alignment in Figure S3a).

The gene with the locus tag GTNG_0206, which is upstream of GTNG_0207 and potentially sharing its operon, codes for a putative RHH domain-harboring antitoxin (Table 1). The aa sequence of the antitoxin is highly similar (~98%) to the antitoxins of the three other *Geobacillus* strains mentioned above.

The antitoxin-coding gene, GTNG_0206, has been annotated as coding for a hypothetical protein in both genome records of the strain (Table S1). The results of our analysis, however, highlighted the identity of this protein as a putative RHH domain-harboring antitoxin (Table 2).

Overall, the MazEF TA family is harbored by all the four *Geobacillus* strains, but three of the strains have two pairs of two different composites; the RHH-MazF and the MazE-MazF, while the fourth one (i.e., *G. thermodenitrificans* NG80-2) has only one pair of the RHH-MazF composite (Table 1 and Table S1). As can be concluded from the gene order and aa sequences, each composite is highly conserved among the analyzed strains (Figures S2 and S3). However, the two composites are diverse (Figures S2 and S3). The antitoxins of the two different composites have different conserved domains, RHH and MazE (Table 1). While the toxin in each composite has a MazF domain, the two composites have two different MazF sequences. The MazF toxins are diverse within one MazF protein family [72]. The MazEF protein pairs of the two composites may coordinate their activity to help the strain adapt to stress [73].

### 2.3. MNT-HEPN

The protein subfamilies MNT (Minimal Nucleotidyltransferase) and HEPN (Higher Eukaryotes and Prokaryotes Nucleotide-binding) form a type II TA pair that is common in archaea and bacteria. While the toxin HEPN is a nucleotide-binding domain that binds to RNA, the antitoxin MNT is a DNA-binding protein that represses the expression of HEPN [74]. Genes coding for HEPN and MNT are highly represented in thermophilic archaea and bacteria [75]. In our analysis, we identified putative MNT-HEPN TA pairs in two strains, each of which has two MNT-HEPN pairs of different composites, as discussed below.

#### 2.3.1. MNT-HEPN (I)—COG1669–COG2361 Composite

##### MNT-HEPN (I) of *Geobacillus* sp. ZGt-1 and *G. thermoleovorans* Gts

The gene encoded on contig 12_20 in ZGt-1 codes for a HEPN-containing protein, as does the gene with the locus tag *_10720 annotated in both genome records of strain Gts. The aa sequences of these proteins are identical (global alignment in Figure S4a) and harbor the domain COG2361 (Clusters of Orthologous Group 2361) which belongs to the DUF86 “domain of unknown function 86” protein family, both of which are associated with HEPN toxin [7,76]. The putative HEPN toxin is labeled here as HEPN (I) in each of the strains (Table 1). 

Most HEPN domains harbor a conserved Rx4–6H catalytic motif (R stands for arginine, H stands for histidine, and x stands for 4–6 of aa residues between R and H), where the residue immediately after the R is a polar aa [77]. This motif is considered the most conserved characteristic of HEPN domains [77]. It is thought to be responsible for the mRNase activity of the HEPN toxins [77]. Both of the above-mentioned HEPN-containing proteins harbor the domain R(75)DMLIH(80), where D is aspartic acid (Asp) which is a polar aa. The presence of this motif further supports the probability that contig 12_20 and *_10720 are part of the putative TA system in those two *Geobacillus* strains.

The gene encoded on contig 12_19, and that with the locus tag *_10710, each of which is adjacent to and upstream of the toxin-coding gene, code for a putative COG1669 domain-harboring protein (Table 1). COG1669 is an MNT antitoxin-associated domain [7]. MNT antitoxin is labeled here as MNT (I) in both strains (Table 1). In strain Gts, there is a slight difference in the aa sequence of the putative antitoxin between the original and RefSeq genome annotations. The putative MNT antitoxin annotated in the RefSeq genome record is perfectly aligned with that of ZGt-1 (Figure S4b). Therefore, we used it for our analysis (Table S1). Nevertheless, in both records, the protein harbors the domain that identifies it as a putative MNT antitoxin. We noticed that the two genes of the TA pair in each strain overlap by 17 nt.

In addition to the MNT and HEPN-coding genes which are adjacent, our analysis indicated that there is another MNT-coding gene potentially sharing the operon with the mentioned putative MNT-HEPN TA pair in each of the strains (Table 1). This second MNT-coding gene in strain ZGt-1 is encoded on contig 12_18, upstream of contig 12_19 and both putative genes are 23 nt apart. This putative MNT (II) also has COG1669 domain (Table 1), and blastp showed that it is 90% identical to nucleotidyltransferase (Table S2). Interestingly, the gene coding for MNT (II) has neither been annotated in the original nor in the RefSeq annotations of the genome records of strain Gts. Therefore, our analysis has identified a gene that has been overlooked and this gene seems to be part of the putative MNT-HEPN TA family of strain Gts. Accordingly, we recommend annotating this MNT-coding gene at the position presented in Table S2. The aa sequences of MNT (II) protein in both strains are identical (Table S1; global alignment in Figure S4c). On the other hand, the aa sequences of the two adjacent MNT (I) and (II) proteins are 19 % identical between the strains ZGt-1 and Gts, respectively. This low matching identity is not unexpected, since nucleotidyltransferase domain-harboring proteins comprise a large and highly diverse protein superfamily [78]. The global alignment confirms that the putative MNT antitoxins of *Geobacillus* strains are diverse (Figure S4b,c). Moreover, our results indicated that *Geobacillus* sp. ZGt-1 and *G. thermoleovorans* Gts represent examples of strains that have putative three-component TA systems among their various TA systems. 

#### 2.3.2. MNT-HEPN (II)–KNTase-COG2445 Composite

The KNTase (kanamycin nucleotidyltransferase) enzyme transfers a nucleoside monophosphate group to aminoglycoside antibiotics, such as kanamycin, leading to the deactivation of the antibiotic. The NTase domain of KNTases is homologous to the MNT domain [76], allowing KNTase to function as an antitoxin within the TA family rather than an aminoglycoside NTase in certain cases [77]. Thus, KNTase and HEPN form a TA pair representing the MNT-HEPN TA family [77]. The COG2445 domain, which belongs to the DUF86 protein superfamily (Table 1), has been identified as a HEPN-associated domain [75]. Therefore, the KNTase-COG2445 composite forms a putative TA pair.

##### MNT-HEPN (II) of *Geobacillus* sp. ZGt-1 and *G. thermoleovorans*Gts

In strain ZGt-1, the aa sequence of the protein encoded on contig 12_83 is identical to that of the protein product of *_11500 (Table S1; global alignment in Figure S5a). These proteins represent putative HEPN (II) toxins, as they harbor the COG2445 domain (Table 1). Moreover, both of the putative HEPN proteins harbor the Rx4–6H catalytic motif mentioned above in the form of R(98)NIAVH(103), where N is a polar aa (aspargine, Asn). These two putative toxins have been annotated as DUF86 domain-containing proteins without specific identification (Tables S1 and S2). Therefore, the current study helped in identifying them as putative HEPN toxins.

In strains ZGt-1 and Gts, each of the upstream genes encoded on contig 12_84 and that with the locus tag *_11510 potentially shares the same two-gene operon with the HEPN (II) coding gene and codes for a putative KNTase-harboring antitoxin (Table 1). The aa sequences of the putative antitoxins are identical (Table S1; global alignment in Figure S5b).

It is likely that both of the KNTase protein products of contig 12_84 and *_11510, in strains ZGt-1 and Gts, respectively, function as putative antitoxins that neutralize the putative HEPN toxin, as was the case for *Shewanella oneidensis* [77]. Here as well, we noticed that the two genes of the putative TA pair in each strain overlap by 11 nt. 

In addition to harboring the R(98)NIAVH(103) motif, the HEPN-containing proteins in both strains harbor E(14)RCLKR(19) that is in line with the EX3KR motif reported by Anantharaman et al., 2013 to be harbored by many HEPN-containing proteins whether they have the Rx4–6H motif or not [76]. However, HEPN-containing proteins are poorly conserved generally [76]. 

Neither *G. kaustophilus* HTA426 nor *G. thermodenitrificans* NG80-2 has the MNT-HEPN TA family.

### 2.4. ParDE–AbrB-ParE Composite

In the canonical type II TA system, the toxin ParE is associated with the antitoxin ParD [79]. ParE is a subfamily of the RelE/ParE toxin superfamily, and it could either have a bacteriostatic or a bactericidal effect, as reviewed in [79,80]. The “mix and match” phenomenon mentioned above applies here as well; in addition to the ParD antitoxin, the ParE toxin may associate with other antitoxins [68,79,81]. For instance, the ParE toxin may associate with the SpoVT-AbrB-type DNA-binding domain (SpoVT stands for Stage V sporulation protein T), as indicated by the NCBI CDD tool. The SpoVT-AbrB domain belongs to the MazE antitoxin superfamily [79,82]. According to the TA domain description, SpoVT-AbrB domain-containing antitoxins are described as AbrB antitoxins [69].

The number of reported AbrB antitoxins is continuously increasing [82]. Although AbrB-ParE has not been reported as a TA composite, we believe we can present it as a ParDE TA family based on the TA family classification by Ou et al., 2013 [69]. According to this classification, a TA pair composed of AbrB-RelE represents RelBE, and since RelE belongs to the same protein superfamily of ParE toxin [83], the association of AbrB with ParE is not unexpected and the TA family could then be named as ParDE. Moreover, in this classification, the TA pair AbrB-Doc represents the Phd-Doc TA family, AbrB-MazF represents the MazEF TA family, and AbrB-PIN represents the VapBC TA family [69]. Our analysis indicated that three of the *Geobacillus* strains have ParDE TA families harboring AbrB-ParE TA composites, as described below.

#### 2.4.1. ParDE of *G. kaustophilus* HTA426 and *Geobacillus* sp. ZGt-1

The gene with the locus tag GK2354, annotated in the RefSeq genome record of strain HTA426, and the one encoded on contig 23_242 in strain ZGt-1 code for a putative ParE-toxin (Table 1). The aa sequences of these two putative toxins are identical between the two strains, except for the start aa, M, which is duplicated in strain ZGt-1 (global alignment in Figure S6a). This duplication also appears in the putative ParE toxin of *G. thermoleovorans* Gts (described below). 

We noticed there are aa sequence differences in the putative toxin between the original and RefSeq genome annotations in strain HTA426. The putative toxin annotated in the RefSeq genome record is better aligned with that of strain Gts (described below). Therefore, we considered it for our analysis (Table S1). Nevertheless, in both genome records, the putative toxin harbors the same ParE toxin domain and has been annotated as a hypothetical protein (Table S1). Contig 23_242 in strain ZGt-1 also codes for a hypothetical protein (Table S2). The current study highlighted the identity of these hypothetical proteins as putative ParE toxins (Table 2).

The analysis also indicated that the gene with the locus tag GK2355, which is adjacent to and upstream of the putative ParE toxin-coding gene annotated in the RefSeq genome record, codes for a putative SpoVT-AbrB domain-containing antitoxin, here labeled as AbrB (I) (Table 1). GK2354 and GK2355 potentially share the operon (Table 1), and overlap by 11 nt. There are also differences in the aa sequences of this AbrB antitoxin between the original and RefSeq genome annotations, but both harbor the SpoVT-AbrB domain. For our analysis, we considered the putative antitoxin annotated in the RefSeq genome record (Table S1). Here as well, the putative antitoxin has been annotated as hypothetical in both genome records (Table S1), but we could identify it as a putative AbrB antitoxin (Table 2). 

In strain ZGt-1, the gene encoded on contig 23_243, which is adjacent to and upstream of the putative ParE-coding gene mentioned above, codes for a putative AbrB (I) (Table 1), with aa sequence that is 100% identical to that of strain HTA426 (Table S1; global alignment in Figure S6b). This antitoxin is 100% identical to a hypothetical protein (Table S2), but the current study identified it as a putative AbrB antitoxin (Table 2). The putative two genes coding for the TA pair of strain ZGt-1, as well, potentially share the operon (Table 1), and overlap by 14 nt.

#### 2.4.2. ParDE of *G. thermoleovorans* Gts

The gene with the locus tag *_26560, annotated in both genome records, codes for a putative ParE toxin (Table 1). The adjacent upstream gene with the locus tag *_26570, annotated in both genome records, codes for a putative SpoVT-AbrB domain-containing antitoxin, AbrB (I) (Table 1). The putative two genes coding for the TA pair potentially share the operon (Table 1), and overlap by 14 nt. Moreover, both genes have been annotated as coding for hypothetical proteins in both genome records (Table S1), but the current study identified them as a putative AbrB-ParE TA pair (Table 2). 

While the aa sequences of the putative ParE toxins and AbrB antitoxins of strains HTA426 and ZGt-1 are (almost) identical, those of strain Gts are shorter and a few of their aa residues are not aligned with these TA proteins in the other two strains (global alignment in Figure S6). 

Our analysis indicated that *G. thermodenitrificans* NG80-2 does not code for the ParDE TA family. 

### 2.5. Phd-Doc–AbrB-Doc Composite

The Phd-Doc family is among the least distributed TA families [50]. In the canonical system, the toxin, Doc (‘‘death on curing”) is associated with the antitoxin, Phd (“prevents host death”) [50]. However, the “mix and match” phenomenon described above has been reported in this TA family as well, where Doc associates with antitoxins other than Phd, and Phd associates with toxins other than Doc [50]. 

The Doc toxin belongs to the Fic (filamentation induced by cyclic AMP) protein superfamily [84] and has a bacteriostatic effect [84,85]. Antitoxins harboring SpoVT-AbrB-like domains that belong to the doc-partner protein family, which shares homology with the SpoVT-AbrB superfamily as indicated by the InterPro domain analysis tool, may associate with the Doc toxin [69]. They form an AbrB-Doc composite, which belongs to the Phd-Doc TA family [69]. *Geobacillus* strains presented here harbor this composite, as discussed below.

#### 2.5.1. Phd-Doc of *G. kaustophilus* HTA426 

The gene with the locus tag GK1846 codes for a protein harboring a Fic/Doc domain (Table 1). Accordingly, the protein product of GK1846 represents a putative Doc toxin.

The gene with the locus tag GK1845 that is adjacent to and upstream of the putative Doc-coding gene potentially shares the operon with GK1846 (Table 1) and the intergenic region between these putative genes is 20 nt long. The domain of the protein product of GK1845 is SpoVT-AbrB-like, which belongs to the “doc-partner” protein family, as indicated by the NCBI CDD and InterPro domain analysis tools. There are differences in the aa sequences of the putative antitoxin between the original and RefSeq genome annotations. Nevertheless, in both records, the protein product harbors the same domain that identifies it as a putative AbrB-like antitoxin, here labeled as AbrB (II) (Table 1). For our analysis, we used the putative antitoxin annotated in the RefSeq genome record as it is better aligned with the putative AbrB antitoxins in the other strains (Table S1; Figure S7b). The annotation of the putative TA pair in the RefSeq genome of the strain confirms our results (Table S1). 

#### 2.5.2. Phd-Doc of *G. thermoleovorans* Gts and *Geobacillus* sp. ZGt-1

The gene with the locus tag *_21530 and that encoded on contig 18_127 in strains Gts and ZGt-1, respectively, code for a protein harboring a Fic/Doc domain (Table 1). Therefore, the encoded two protein products represent putative Doc toxins. Their aa sequences are identical between the two strains (Table S1; global alignment in Figure S7a). The NCBI annotation of these two proteins confirms our results (Table S1). 

The analysis also indicated that the gene with the locus tag *_21520 and that encoded on contig 18_126, both of which are adjacent to and upstream of the Doc-coding gene in the two strains, code for a putative protein harboring a SpoVT-AbrB-like domain, which belongs to the “doc-partner” protein family. Therefore, these protein products represent putative AbrB (II) antitoxins (Table 1). Their aa sequences are 99% identical (global alignment in Figure S7b), and their NCBI annotations also confirm our results (Table S1). The intergenic region between the putative TA pair genes is 20 nt long in each of the strains.

Overall, the aa sequences of the putative Doc toxins of the three *Geobacillus* strains discussed above are (almost) identical (global alignment in Figure S7a). While the aa sequences of the putative AbrB (II) antitoxins of strains Gts and ZGt-1 are almost identical, that of strain HTA426 is shorter than the AbrB (II) antitoxins of these other two strains, and a few of its aa are not aligned (global alignment in Figure S7b).

Our analysis indicated that *G. thermodenitrificans* NG80-2 does not code for the Phd-Doc TA family. 

### 2.6. RelBE–XRE-RelE Composite

RelBE is one of the best-described TA families [86]. In the canonical system, the toxin, RelE (Relaxed E) is associated with the antitoxin, RelB (Relaxed B) [9,86]. However, the “mix and match” phenomenon also applies, where the RelE may associate with other antitoxins such as AbrB- and RHH-domain harboring proteins, XRE (Xenobiotic Response Element) family proteins, and Phd antitoxin [69]. We are presenting the XRE-RelE TA composite, which belongs to the RelBE TA family [69], since it is the one found in the strains analyzed here (for more information on the RelB antitoxin and the other possible composites, the reader is referred to [86]).

RelE and ParE toxins both belong to the ParE superfamily [86]. RelE has a bacteriostatic effect [17]. The XRE protein family is a large family of transcriptional regulators, with an HTH DNA-binding motif, that controls different functions in prokaryotic cells [75,87]. The *xre* gene regulates the transcription of its own gene as well as other neighboring genes [88]; therefore, it has the potential to function as an antitoxin. Moreover, XRE proteins can inactivate toxins [89]. Since proteins functioning as antitoxins are not necessarily specialized in functioning that way exclusively, XRE proteins may act as antitoxins [75,90] that pair with e.g., RelE toxin [7,8,75,86,91,92]. Three of the four *Geobacillus* strains have a putative XRE-RelE TA composite, as discussed below.

#### 2.6.1. RelBE of *G. kaustophilus* HTA426 and *Geobacillus* sp. ZGt-1

The gene with the locus tag GK3104, annotated in both genome records of strain HTA426, and the gene encoded on contig 25_195 in strain ZGt-1 code for a putative RelE domain-harboring protein (Table 1). Therefore, it is likely that each of these two genes codes for a putative RelE toxin. The aa sequences of these two putative toxins are 99% identical (global alignment in Figure S8a).

The gene with the locus tag GK3105 in strain HTA426, and the one encoded on contig 25_196 in strain ZGt-1, each of which is adjacent to and upstream of the putative RelE toxin-coding gene, seem to code for a putative antitoxin. The two genes coding for each of the putative TA pairs in each of the strains overlap by 32 nt. In strain HTA426, the two putative genes potentially share the operon (Table 1). Although the Operon-Mapper did not annotate the genes encoded on contigs 25_195 and 25_196 in strain ZGt-1, it could be easily inferred that the two putative genes potentially share the operon (Table 1). 

The aa sequences of the protein products of GK3105 and contig 25_196 are 98% identical (global alignment in Figure S8b). Neither the NCBI CDD nor the InterPro domain analysis tools identified a conserved domain in the protein products of GK3105 or contig 25_196. GK3105 has been annotated as a hypothetical protein in its genome records (Table S1), and contig 25_196 is 99% identical to a hypothetical protein (Table S2). However, when checking the orthologs of GK3105 presented in the KEGG database, we found that GK3105 is orthologous to a *G. genomosp.* strain 3 protein, AGT33452, and showed 100% identity over its entire length. AGT33452 is annotated as an XRE family transcriptional regulator in the genome of *G. genomosp.* strain 3 [93]. Moreover, among the other orthologs that are annotated as XRE family transcriptional regulators are ALA70040, AMQ22632, and AMX82334, all of which are geobacilli proteins that showed ~99%, ~98%, and ~94% identity to GK3105, respectively. The protein encoded on contig 25_196 in strain ZGt-1 is also orthologous to the same proteins, ALA70040, AGT33452, AMQ22632, and AMX82334, which showed ~98%, ~97%, ~97%, and ~96% identity, respectively. However, each of these four proteins has been annotated as hypothetical in its RefSeq version. On the other hand, when searching for proteins homologous to GK3105 and the protein encoded on contig 25_196 within *Bacillus* species, using blastp, the best hit was “XRE family transcriptional regulator” (WP_066367164) of *B. fumarioli* NBRC 102428, which showed 56% identity to the two geobacilli proteins, with an e-value of 9e-27 and query coverage of 97% (GK3105), and an e-value of 6e-25 with a query coverage of 98% (contig 25_196). It is worth noting that, as is the case with GK3105 and the protein encoded on contig 25_196, this “XRE family transcriptional regulator” of *Bacillus* has no conserved domain, as indicated by the NCBI CDD and InterPro domain analysis tools. Moreover, the secondary structure prediction of the two geobacilli XRE proteins showed that they have repeated alternation between coils and helices, similar to WP_066367164 (Figure S11a–c).

Taken together, these results indicate that the protein products of GK3105 and contig 25_196 could represent putative XRE family transcriptional regulators acting as antitoxins (Table 2). Experimental studies are required to confirm the identity of these geobacilli proteins. 

In summary, protein products of GK3105 and GK3104, and those of contigs 25_196 and 25_195 represent putative XRE-RelE TA pairs (Table 1).

#### 2.6.2. RelBE of *G. thermoleovorans* Gts

The gene with the locus tag *_34810 codes for a putative RelE domain-harboring protein (Table 1). Therefore, it is likely that this gene codes for a putative RelE toxin. *_34820, which is upstream of and overlapping with the putative RelE-coding gene by 32 nt, most likely codes for a putative antitoxin. The putative antitoxin encoded by the gene locus *_34820 lacks a conserved domain, as is the case with those of strains HTA426 and ZGt-1, and it has been annotated as a hypothetical protein in both of the strain genome records (Table S1). However, it is orthologous to the same “XRE family transcriptional regulator” proteins mentioned above, AGT33452, A0V43_13420, ALA70040, AMX82334, to which it is ~99%, ~99%, ~98%, and ~93% identical. Moreover, the NCBI blastp results showed that the protein product of *_34820 is 55% identical to the “XRE family transcriptional regulator” (WP_066367164) mentioned above, with an e-value of 4e-26 and query coverage of 97%. Furthermore, as is the case with the other two geobacilli strains mentioned above, the secondary structure of the putative XRE protein in this strain has repeated alternation between coils and helices (Figure S11d). These results indicate that the protein product of *_34820 could as well represent a putative XRE family transcriptional regulator acting as an antitoxin (Table 2). 

According to our analysis, the protein products of *_34820 and *_34810 represent a putative XRE-RelE TA pair (Table 1).

While the aa sequences of the putative RelE toxins and XRE antitoxins are 99% and 98% identical, respectively, in strains HTA426 and ZGt-1, the aa sequence of the putative RelE toxin of strain Gts is 92% identical to that of strain HTA426 and 93% identical to that of ZGt-1, and the aa sequence of the putative XRE antitoxin is ~98% and ~96% identical to the putative XRE antitoxins of strains HTA426 and ZGt-1, respectively (Figure S8). 

Our analysis indicated that *G. thermodenitrificans* NG80-2 does not code for the RelBE TA family. 

### 2.7. VapBC–COG2886-PIN Composite

VapBC (virulence associated protein) is the most widespread TA family in bacteria and archaea [94], but the least well-described [95,96]. The VapBC family is composed of a protein harboring a PIN domain as the toxin, and a DNA-binding domain as the antitoxin [95]. 

The PIN (PilT N-terminus) domain, is a type of pili protein [95] associated with the ribonuclease activity of the VapC toxin, which has a bacteriostatic effect [94,97]. The PIN-like domain-harboring toxin could associate with any protein harboring a DNA-binding domain as an antitoxin [95]. Different TA composites have been reported [22,96], one of which is the COG2886-PIN composite [69]. COG2886 proteins belong to the UPF0175 (uncharacterized protein family 0175) protein superfamily, according to the NCBI CDD analysis tool, and represent putative DNA-binding antitoxins [75,90]. Only two of the four *Geobacillus* strains have a putative VapBC family, the COG2886-PIN composite, as discussed below.

#### VapBC of *G. kaustophilus* HTA426 and *G. thermoleovorans* Gts

The genes with the locus tags GK1949 and *_22480 in HTA426 and Gts, respectively, code for a putative COG2405 domain-harboring protein, belonging to the DUF3368 protein family (Table 1). The COG2405 is a PIN-like domain, and thus the protein is a putative VapC toxin [90]. The aa sequences of these two putative toxins are identical between the two strains (Table S1; global alignment in Figure S9a). In both strains, we noticed that there are differences in the aa sequences of the putative toxins between the original and RefSeq genome annotations. Nevertheless, in both records, the protein harbors the same domain that identifies it as a putative VapC toxin. For our analysis, we used the putative toxin annotated in the RefSeq genome record of each strain (Table S1).

The genes with the locus tags GK1950 and *_22490 code for a putative antitoxin harboring the domain COG2886 (Table 1). The aa sequences of these two putative antitoxins are identical between the two strains (Table S1; global alignment in Figure S9b). Each of the putative coding genes is adjacent to and upstream of the toxin-coding gene. The coding genes are also annotated differently in the RefSeq and the original genome records of each strain. Nevertheless, in both records, the protein product harbors the same putative antitoxin-associated domain, the COG2886. For our analysis, we used the antitoxin annotated in the RefSeq genome record (Table S1). The two genes coding for each of the putative TA pairs potentially share the operon in each of the strains (Table 1), and overlap by eight nt.

Neither *G. thermodenitrificans* NG80-2 nor *Geobacillus* sp. ZGt-1 has VapBC TA system.

### 2.8. XRE-COG2856

The XRE-COG2856 is a potential novel TA family [98]. It was discovered in 2009 by Makarova et al. based on an in-silico analysis and found to be abundant in the genomes of bacteria, archaea, and phages [75,98]. This TA family has not been experimentally characterized yet [94]; therefore, it is still unknown whether it represents a functional TA family or not [8].

The putative toxin, harboring the COG2856 domain, is a protease that belongs to the metzincin Zn-dependent proteases [75], and is part of the DUF955 protein superfamily, according to the NCBI CDD tool. Metzincin Zn-dependent proteases, including COG2856-harboring proteins, are recognized by having a conserved HEXXH motif as the Zn-binding catalytic active site, where X is any aa [75]. 

The COG2856-harboring toxin is usually accompanied by an HTH domain-harboring protein of the XRE-family, acting as the antitoxin [75]. Often, the HTH-domain is fused with the COG2856 domain in a single protein [75,98], but this does not seem to be the case with the strains analyzed here, as discussed below.

#### 2.8.1. XRE-COG2856 of *G. kaustophilus* HTA426 and *G. thermoleovorans* Gts

The genes with the locus tags GK3184 and *_35620 in strains HTA426 and Gts, respectively, code for a putative COG2856 domain-harboring toxin (Table 1). The aa sequences of the putative toxins are >99% identical between these two strains (global alignment Figure S10a). The sequence of each putative toxin contains the HEXXH motif as HEFYH. The NCBI annotation supports our results, as it shows that each of these putative proteins is identical to a member of the ImmA/IrrE metallo-endopeptidase family (Table S1), which consists of Zn-dependent proteases harboring the COG2856 domain mentioned above [99,100]. However, the current study further clarified that the two proteins are putative toxins of the type II TA system.

The genes with the locus tags GK3185 and *_35630, which are adjacent to and upstream of the putative toxin-coding gene, code for a putative protein that does not harbor a conserved domain, as indicated by the NCBI CDD and InterPro domain analysis tools (Table 1). However, the protein motif presented in the KEGG database showed that the protein products of GK3185 and *_35630 harbor the HTH domain (e-value < 0.02). Therefore, they might represent putative antitoxins of the XRE-protein family. The aa sequences of the two putative antitoxins are ~98% identical between these two strains (Figure S10b). They have been annotated as hypothetical proteins in the genome records of each strain (Table S1). The current study, however, highlighted the identity of these hypothetical proteins as putative antitoxins (Table 2).

In each strain, the intergenic region between the putative TA pair-coding genes is 4 nt long. This unfused pattern of COG2856-XRE does not seem to be common among prokaryotes, as indicated in [75], or it could simply be harbored by yet to be studied strains. The putative coding genes potentially share the operon with a third gene, locus tags GK3183 in strain HTA426, and *_35610 in strain Gts, positioned downstream of the TA-coding genes. This third putative gene codes for a hypothetical protein in each strain (Table S3). Analysis of the aa sequence using the NCBI CDD and InterPro domain analysis tools did not retrieve a protein domain (Table 1). However, the protein motif presented in the KEGG database showed that the protein products of GK3183 and *_35610 harbor the protein domain 7TMR-HDED (e-value < 0.26). The 7TMR-HDED stands for 7 transmembrane helices receptors-HD hydrolase; a hydrolase with a catalytic His-Asp (HD) motif, and ED stands for extracellular domain [81,101]. The pfam of the 7TMR-HDED protein family is PF07697, as indicated by the NCBI CDD and InterPro domain analysis tools. The 7TMR-HDED domains are expected to be involved in signal detection and transmission to the cellular machinery, in order to stimulate a response to the environmental conditions [81,101]. The presence of the 7TM-HDED receptor suggests that the protein product of GK3183 is regulated by a second messenger, which is likely to be the c-di-AMP, since the 7TMR domains transmit the c-di-AMP [97], and in turn, it regulates other proteins. 

We analyzed the genome context to have a further indication of the kind of second messenger that the two “hypothetical” proteins transmit, and thus could affect the TA pair, since it has been reported that genes within the neighboring context have provided information on novel signaling nucleotides [81]. We found that a gene with the locus tag GK3182 in strain HTA426, and another with the locus tag *_35570 in strain Gts are involved in the adenine metabolism, as shown in the KEGG pathway maps of the purine metabolism in each of the strains, and have been annotated in the RefSeq genome record of the respective strain, as well as in the KEGG database, as “bifunctional 2’,3’-cyclic-nucleotide 2’-phosphodiesterase/3’-nucleotidase (EC:3.1.4.16 3.1.3.6)”. This type of enzyme is expected to function as a phosphodiesterase acting on specific cyclic di-nucleotides, not on the cyclic nucleotide monophosphate—as was the case with the CdnP, whose annotation is the same as the protein products of GK3182 and *_35570, but was experimentally proved to hydrolyze the c-di-AMP [102]. Taken together, we could assume that the signal which the 7TMR domain of GK3183 and *_35610 transmits is the c-di-AMP.

The c-di-AMP signaling molecule, a recently discovered second messenger [103], is synthesized by many bacteria and archaea [104]. Among firmicutes, the c-di-AMP was found essential for the *B. subtilis* growth; and thus, it is the only essential signaling nucleotide reported so far [104]. However, it was found to be an essential signaling nucleotide for the growth of *Listeria monocytogenes* [105] and *Staphylococcus aureus* [106] only under specific growth conditions. Such examples must be considered when experimentally studying the c-di-AMP in a given bacterial strain. The synthesized c-di-AMP molecules are secreted into the extracellular space [101,102], and this secretion is possibly related to stress responses [107].

The presence of a protein that senses the c-di-AMP molecule in the same operon with the genes coding for the TA pair is unlikely to be a random incidence. There is possibly a functional link between the c-di-AMP and the COG2856-XRE TA pair, especially since several studies reported that the two signaling messengers, c-di-AMP and (p)ppGpp are interconnected via an unknown mechanism [101,104,108,109,110]. The signaling messenger, (p)ppGpp, also known as the “stress messenger” [111], mediates the stringent response, which is the response that allows bacteria to adapt to stresses, by coordinating different biological processes [108,112]. The activation of the toxins of TA systems is a part of the stringent response activated by the (p)ppGpp [22,112].

Since stress conditions require the release of bacterial communication signals for regulating the response [113], it is not surprising that the regulation of the TA modules is connected to the cellular signaling pathways [3]. Toxins are target-specific; thus, they are connected to specific cell signals that control their activation [3]. So far, the signal that has been widely reported to control the toxin activation is the (p)ppGpp. High levels of the (p)ppGpp inhibit the exopolyphosphatase (PPX) [22]. This, in turn, leads to the accumulation of the polyphosphate (PolyP) [22]. The PolyP is a signaling molecule that activates the Lon protease, which degrades the antitoxin, resulting in the release of the active toxin [22]. Accordingly, high levels of (p)ppGpp eventually lead to the toxin activation. Since, the two messengers (p)ppGpp and c-di-AMP have a bidirectional relation, as mentioned above [101,104,108,109,110], the conditions that raise the (p)ppGpp levels will also raise those of the c-di-AMP.

**Figure 2:**
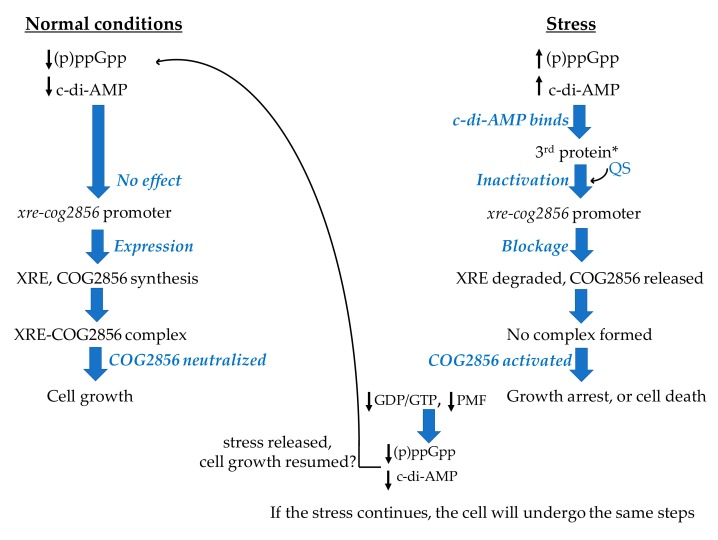
Scheme illustrating the hypothesis proposed in the current study on the regulation of the *xre*-*cog2856* expression by the signaling messengers, (p)ppGpp and c-di-AMP. * 3^rd^ protein represents the protein products of GK3183 and *_35610. QS stands for quorum sensing molecule(s). The black up arrows indicate increased levels, while the black down arrows indicate decreased levels. The small black curved arrow indicates the involvement of the QS molecule(s). The big black curved arrow indicates that the cell could go back to normal conditions. Scheme inspired by a figure in Gross et al., 2006 [111].

Based on the roles of the c-di-AMP and (p)ppGpp in controlling cellular processes under unfavorable conditions and the reported crosstalk between them, we are proposing a hypothesis for the regulation of the *cog2856-xre* TA gene expression by these two signaling molecules (Figure 2). We are first presenting the hypothesis and following up with findings of previous studies that we based our hypothesis on.

#### 2.8.2. Hypothesis—Regulation of the *xre-cog2856* Expression

As illustrated in Figure 2, when bacterial cells are experiencing stress, (p)ppGpp levels increase, and this in turn will raise the c-di-AMP levels, since they are interconnected as mentioned above. The opposite may also take place, where the c-di-AMP level could rise first and the increase in (p)ppGpp level would follow. The c-di-AMP synthesis from ATP is catalyzed by the diadenylate cyclase (EC:2.7.7.85) [114], while the (p)ppGpp synthesis from ATP and either GTP or GDP is catalyzed by the enzyme RSH (a bifunctional RelA/SpoT homologue) (EC:2.7.6.5) [112]. These two signaling messengers help the cells cope with the stress through synchronizing cellular responses [110]. The synthesized c-di-AMP molecules would be secreted into the extracellular space and would be sensed by the protein product of GK3183 in strain HTA426 and the product of *_35610 in strain Gts via the extracellular receptor domain (7TMR-HDED) mentioned above. The GK3183/*_35610 protein might then experience conformational changes and could function as a regulatory protein that negatively regulates the expression of the adjacent TA loci. This regulation could take place via one or more quorum sensing molecules that, under stress, could lead to the inhibition of the TA gene expression. Therefore, the synthesis of the TA pair, together with GK3183/*_35610, would stop, and this would result in activating the toxin, since the unstable antitoxin would have been degraded by the Lon protease (EC:3.4.21.53), which is activated by the high level of (p)ppGpp [112]. Consequently, the cell growth would be halted. When the conditions improve, the levels of (p)ppGpp and c-di-AMP would decrease and the expectedly stable GK3183/*_35610 protein would sense that and unblock the expression of the TA-coding genes via one or more quorum sensing molecules. The cell could then resume its growth. Since the mechanism of action of the toxin COG2856 is still unknown, it cannot be confirmed whether the cells will resume their growth when the conditions improve or not. The cells could undergo the PCD after reaching the “point of no return” [66]. 

The genes coding for the enzymes needed for the synthesis of the two signaling messengers and for the Lon protease have been annotated on the genomes of the two strains. The locus tags of the gene coding for the diadenylate cyclase (EC:2.7.7.85) are GK0152 and *_1680, those coding for the RSH enzyme (EC:2.7.6.5) are GK0829 (and GK2578) and *_29020 (and *_9850), and those coding for the Lon protease are GK2650 and *_29780 in strains HTA426 and Gts, respectively. 

The regulation of the expression of TA-coding genes could take place at the transcription level, the translation level, or both [115]. In the case of COG2856-XRE, we expect the regulation to be at the transcription level since the (p)ppGpp is a transcription regulator [112], as is the c-di-AMP [107].

We have envisioned our hypothesis based on findings of previous studies, as discussed below. The c-di-AMP level is crucial for the cell [104]. In Gram positive bacteria, intracellular levels of c-di-AMP are expected to change in response to environmental or intracellular signals [109,116]. Neither the causes nor the exact results of changes in c-di-AMP levels and their influence on the whole cell physiology are well understood [110]. After sensing a stimulus raising the c-di-AMP level, the latter transduces the signal by binding to a receptor or a protein and changing its conformation [109,116,117]. This in turn will trigger a signal cascade, resulting in the regulation of different cellular processes, including the gene expression [109,116,117]. Accordingly, the regulation of the TA gene expression may possibly be one of the c-di-AMP targets. The regulation of type II TA system expression by a receptor protein of a second messenger, cyclic AMP, has been reported along with speculation on the involvement of quorum sensing molecules in the regulation [118]. The possibility of regulating the TA expression via quorum sensing has also been reported in other studies [5,119]. Expanding on this, we could assume that the regulation of *xre*-*cog2856* expression by the second messenger, c-di-AMP, via quorum sensing is possible.

The high level of c-di-AMP molecules activates the (p)ppGpp-synthesizing enzyme via an unknown mechanism, causing an increase in the level of (p)ppGpp [108,110]. On the other hand, independently of c-di-AMP, the (p)ppGpp level also increases due to environmental stresses [112]. The increase in the level of (p)ppGpp affects different cellular processes in order to control the growth rate, including the inhibition of the ribosomal RNA (rRNA) synthesis, regulation of nucleotide synthesis, and differential gene transcription to help bacteria adapt to stress [112]. The high level of (p)ppGpp strongly inhibits GTP synthesis, which in turn inhibits protein biosynthesis [112,120,121]. Moreover, the high level of (p)ppGpp could inhibit the expression of TA promoters, leading to toxin activation as a measure to help the bacterial population survive the stress, such as the case reported for the MazEF promoter in *E. coli* [12,111]. Furthermore, high levels of (p)ppGpp prevent the degradation of c-di-AMP, leading to the accumulation of the latter, which thus becomes toxic to the cell, as was shown for *B. subtilis* [104,122] (for details on the effects of (p)ppGpp on cellular processes and gene transcription in bacteria, the reader is referred to [112,123,124]). Conclusively, regardless of which one of the two signaling messengers increases first, the level of the other one will increase as well. Consequently, the cells will be left under high levels of both messengers, and their elevated levels constitute a stress signal that the cells respond to in the ways mentioned above. 

In addition to the potential role of the high levels of these two messengers in regulating the expression of the TA promoter, they could act as mediators that enhance the toxin-induced effects in order to create a rapid response, due to their actions mentioned above. However, their role in enhancing the growth inhibition will be limited. Since (p)ppGpp is synthesized from GTP/GDP and since the high level of (p)ppGpp inhibits salvaging and de novo synthesis of these nucleotides [120,121,125,126], the synthesis of further (p)ppGpp will cease. The same applies to c-di-AMP since metabolically inactive cells have a reduced proton motive force (PMF) (i.e., reduced ATP level) [24,127]. The cessation of further formation of the two messengers could protect the cell from “early” death, with the hope that the conditions may improve. When the stress is released, (p)ppGpp and c-di-AMP levels will be reduced to basal levels; therefore, the TA promoter will be unblocked. If the toxin has had only a bacteriostatic effect, or if the cells have not reached the “point of no return” mentioned above, the cells will recover and resume their growth.

The regulation of a TA pair by a third protein encoded by a gene downstream of the TA-coding genes and potentially sharing the same operon has been reported previously for the MazEF pair in *E. coli* [111]. The principle of delaying cell death has also been reported in the same study [111]. Additionally, the existence of an interconnection between a type II toxin and second messengers, the (p)ppGpp and the c-di-AMP has been reported previously in [24]. Álamo et al. have reported an interrelationship between a type II toxin (ξ), the (p)ppGpp, the c-di-AMP, and other nucleotides [24]. However, the effects of these two messengers on inducing the TA expression were not studied since the expression was controlled by external inducers [24]. Taken together, the conclusions of the mentioned studies support the basis of our hypothesis.

In summary, we identified an uncommon pattern of unfused genes coding for the XRE-COG2856 TA pair in *G. kaustophilus* HTA426 and *G. thermoleovorans* *_35610. We highlighted for the first time the possible roles of the c-di-AMP and (p)ppGpp in regulating the *xre-cog2856* expression and the toxin activity. This will pave the way for experimental investigation of this TA pair. 

Understanding the mechanisms of interaction between c-di-AMP and (p)ppGpp messengers and their role in coordinating stress responses in *Geobacillus* strains will broaden our knowledge, especially since the field of c-di-AMP signaling in bacteria has emerged only recently and is still growing.

Analysis of the draft genome of *Geobacillus* sp. strain ZGt-1 showed that it does not code for XRE-COG2856. The genes coding for this TA pair could be encoded on the part of the genome that has not been sequenced, or it could simply be that the strain does not code for this TA pair. *G. thermodenitrificans* NG80-2 harbors the genes coding for the XRE-COG2856, but these putative genes are within the region of the prophage [128]; thus, they are beyond the scope of this study.

### 2.9. TAs of *Geobacillus* Strains—Conserved, Yet Diverse

TA families of every *Geobacillus* strain analyzed here are highly diverse (Figures S1–S10); it was not possible to get a consensus when a global alignment of all sequences was tried. Moreover, in a strain that has more than one toxin/antitoxin molecule belonging to the same TA family, diversity is observed among these molecules, such as the observations reported here about the MazEF and MNT-HEPN TA pairs and the MNT solo antitoxins as well (Figures S2–S5). However, for each TA family, the TA pairs are highly conserved among the four strains, but TAs of *G. thermodenitrificans* NG80-2 are much lower in number and are relatively less conserved compared to the other three strains (Figures S1 and S3). One of the reasons could be related to the different ecological niches where the strains were isolated from. Strain NG80-2 was isolated from an oil field [32], while the other three strains were isolated from aquatic environments [27,30,31]. 

The nature of the environment may also have an important impact on the number of encoded TAs, as has been suggested for archaea [129,130]. Terrestrial hot springs (such as Ulu Slim, where *G. thermoleovorans* Gts was isolated from, and Zara, where *Geobacillus* sp. ZGt-1 was isolated from) represent challenging environments for the cells due to continuous fluctuations in temperature over a wide range, along with changes in nutrient levels and pH [129]. This may explain the reason why strain Gts harbors the highest number of TA loci, 10 putative TA pairs and one apparently solo putative antitoxin, among the four strains. Strain ZGt-1 has eight putative TA pairs and one apparently solo putative antitoxin, but since its genome sequence is incomplete, it cannot be determined whether these are all the type II TA loci that strain ZGt-1 harbors, or it does have more.

On the other hand, there does not seem to be a correlation between the temperature of the ecological niche of the strain and the number of TA loci. A known example showing the lack of this correlation is illustrated by *Mycobacterium tuberculosis*, which is a mesophilic species and its strains harbor up to 67 type II TA pairs [131]. Regarding the *Geobacillus* strains analyzed here, while the temperature of the Dagang oil field (strain NG80-2) at the time of isolating the strain was 73 °C [32], the strain has only two putative TA pairs. Contrarily, the temperature of the Mariana Trench and Zara hot spring were 55 °C [30] and 46 °C [27], respectively, and strains HTA426 and ZGt-1 have eight putative TA pairs each. Moreover, the temperature of Ulu Slim at the time of isolating strain Gts was around 92 °C and the strain has 10 TA pairs, as mentioned above [31]. Experimental analysis is needed to conclude the impact of different environmental factors [129].

It is worth noting that in an exceptional case of a TA pair of the type I TA system in *E. coli*, the toxin SymE (SOS-induced *yjiW* gene with similarity to *MazE*) of the SymE-SymR TA pair showed homology to the MazE antitoxin, as it harbors an AbrB domain [132]. However, experimental results indicated that SymE is actually a toxin that seems to have evolved from the AbrB-domain protein superfamily [132]. Accordingly, such exceptions should be kept in mind when carrying out experiments, especially when studying solo toxins and antitoxins, since an antitoxin domain-harboring protein could be a toxin that evolved from an antitoxin protein family, or vice versa. As is the case with any protein and as mentioned above, only experiments may confirm the function of a predicted toxin/antitoxin.

### 2.10. Applications of TAs

#### 2.10.1. The Potential of TAs as Antibacterial Agents—Pharmaceutical Industry

Toxins of the TA systems constitute an attractive source of antibacterial drugs due to their bacteriostatic and bactericidal effects [5]. They could most likely be used as “standalone” antibacterial agents or combined with one of the conventional antibiotics to generate a synergistic antibacterial effect [5]. In either case, this will require a thorough understanding of the functionality of the toxin to make it druggable, including the interaction between the multiple copies of the same toxins, as well as understanding the interaction between the closely related antitoxins harbored by the strain [5]. As is the case with developing any novel drug, there are certain requirements that the combination of the toxin and conventional antibiotic, or the toxin to be drugged must meet (for details, the reader is referred to [5,133]. Engineering the toxin protein might be a successful approach to enhance its antibacterial activity, eliminate its harmful effects on human cells, and increase its stability in the human serum, as was demonstrated by Solecki et al., 2015 [134]. Since *Geobacillus* strains have several TA pairs, it could be of interest to investigate their potential as a source of thermostable antibacterial drug candidates.

#### 2.10.2. The Potential of *Geobacillus* TAs as Antibacterial Agents and Antibacterial Targets—Food Industry

Toxins of *Geobacillus* strains could represent potential antibacterial candidates that antagonize the growth of geobacilli which cause problems in the food industry. The thermophilic *G. stearothermophilus* is a known food-spoiling bacterium. The cells create biofilms on the stainless steel of the processing lines in dairy and food factories, and thus spoil the final product [135]. In a previous study, we demonstrated that *Geobacillus* sp. ZGt-1 antagonized the growth of a strain of *G. stearothermophilus* via the production of antibacterial proteins [27]. Similarly, TA system toxins of strain ZGt-1 or any other *Geobacillus* strains could be exploited as antibacterial agents to antagonize the growth of *G. stearothermophilus* in dairy and food factories.

On the other hand, TA systems could be a target for toxin-activating molecules. By running a quick search for type II TA genes of *G. stearothermophilus* DSM 458, using the TA finder tool, we found the strain harbors many TA pairs belonging to different type II TA families. As mentioned above, cells of *G. stearothermophilus* form biofilms, and this feature could be due to the harbored TA genes (reviewed in [47]). Deletion of the strain TA genes could solve the problem of biofilm formation, as was shown for *E. coli* (reviewed in [5]). 

Accordingly, in a factory where *G. stearothermophilus* is causing problems, sequencing the genome of the strain and identifying the putative TA genes using bioinformatic tools, followed by identifying the functional ones experimentally, then choosing the TA to be targeted and selecting or designing one or more molecules that can activate the toxin, inactivate the antitoxin or disrupt the TA complex could be an effective approach for eliminating the strain capability of forming biofilms.

An overview of the several potential applications of TA systems is available in [136].

## 3. Materials and Methods 

### 3.1. Identification of TA Pairs Using the TA Finder

For the identification of the TA pairs of the three *Geobacillus* type strains, *G. kaustophilus* HTA426, *G. thermodenitrificans* NG80-2, and *G. thermoleovorans* Gts, the “predict” tool of the TA finder version 2.0 [25] was used. The chromosome and plasmid sequences of the type strains were selected from the available complete genome list and then analyzed. For the non-type strain, *Geobacillus* sp. ZGt-1, annotation of its draft genome sequence was carried out first, using CDSeasy gene prediction and functional annotation tool [137] recommended by the developers of the TA finder. The genome sequence was annotated by Prodigal [138] and then uploaded into the TA finder, where default parameters were used to mine the genome for TA pairs.

### 3.2. Analysis of the Identified TA Pairs

#### 3.2.1. NCBI BLAST Analysis

The TA finder-predicted TA sequences were subjected to the NCBI blastp (2.8.1+) [139] against the RefSeq database, where default settings were used and the resulting e-values were equal (or very close) to zero, and the RefSeq and original genome records of each strain were also manually inspected for each TA pair sequence. The annotation of the TA-coding genes, the protein description, and the nt and aa sequences in the genome records were checked. Whenever there was a discrepancy in the annotation between the RefSeq and original genome records, the annotations of the RefSeq records were selected except for the TA pair GK1498 and GK1499, where the toxin of GK1498 of the original record was better aligned with the other toxins of the same TA family. However, in discrepancy cases, the protein domain analysis (see below) was carried out for the protein sequence of each record in order to confirm that in both records, the protein has a toxin-/antitoxin-related domain, as explained in the main text. For the antitoxin MNT (II) sequence which is encoded by an unannotated gene that we identified using the Operon-Mapper (see below), we ran the tblastn (2.8.1+) [139] against the Nucleotide collection database and retrieved the nt sequence, which was identical to that predicted by Operon-Mapper, and also retrieved the gene position (Table S2). Moreover, the aa sequences of the proteins whose coding genes share the operon with the TA-coding genes were analyzed using the NCBI blastp (2.8.1+), and following the criteria mentioned above.

#### 3.2.2. Protein Domain Analysis—CDD and InterPro Tools

The protein domain of a given toxin/antitoxin aa sequence was identified using the NCBI CDD tool [140] and the InterProScan sequence search tool [141]. These tools were used to confirm that the TA predicted proteins harbor toxin-/antitoxin-related domains. 

#### 3.2.3. Alignment of TA Sequences

TA aa sequences of each strain were used as input to Proteinortho (version 5) [142] using default settings, and the output was then used to split all TA sequences, one sequence file per reported group. The faa file of each protein group was then run through Clustal Omega (version 1.2.4) [143], using default settings. The TeXshade package [144] was used to visualize the alignments by converting the multiple sequence alignments to color images, using the “similar” shading mode with the “all match special” option.

#### 3.2.4. Operon Prediction

The putative operon for every TA pair-coding gene was predicted using the Operon-Mapper [35], where default settings were used. The RefSeq genome records of all type strains and the draft genome of strain ZGt-1 were uploaded and operons of TA pairs were identified. The Operon-Mapper also helped in identifying genes, and aa sequences of their protein products, that share the operon with certain TA pairs (Table S3). The protein products of these genes were analyzed for their domains and the identified TA-related proteins and their genes were selected to be further analyzed following the same steps used for analyzing the TA finder-predicted TAs (Figure 1). For the three type strains, when the Operon-Mapper did not predict the operon of a certain TA pair, the Prokaryotic Operon DataBase (ProOpDB) (http://biocomputo2.ibt.unam.mx/OperonPredictor/) [36] was used instead. 

#### 3.2.5. KEGG Database

The KEGG Genes database [34] was used to retrieve the orthologs and the protein motifs of certain genes/proteins in certain type strains. The KEGG Pathway database was used to retrieve the purine metabolism in specific type strains, as discussed in the main text. The BLAST KOALA tool was used to identify orthologs of the gene encoded on contig 25_196 of *Geobacillus* sp. strain ZGt-1. The protein sequences of this strain were uploaded to BLAST KOALA (version 2.1) [34], where “Bacteria” was selected as the taxonomy group, and the BLAST was done against the “species_prokaryotes” KEGG Genes database.

#### 3.2.6. Secondary Structure Prediction

The PSIPRED workbench tool [145] was used.

## 4. Conclusions

In this study, we conducted an in-silico genome analysis for the identification of bacterial TAs in 4 *Geobacillus* strains; *G. kaustophilus* HT426, *Geobacillus* sp. ZGt-1, *G. thermodenitrificans* NG80-2, and *G. thermoleovorans* Gts. The analysis was carried out by employing the TA finder software and the resulting sequences were manually curated using the NCBI CDD and InterPro domain analysis tools, as well as inspecting both genome records of each strain (Figure 1).

We identified 28 putative TA pairs, distributed over eight TA families (Table 1), potentially targeting various cellular processes, in the 4 strains. Out of the identified putative TAs, 15 represent putatively novel TAs (Table 2). We found that the number and families of type II TA pairs varied among the four strains. While *G. thermoleovorans* Gts has the eight TA families that were found in all the four strains with a total number of 10 putative TA pairs and one apparently solo putative antitoxin, *G. thermodenitrificans* NG80-2 has only two TA families, with two TA pairs, and two and one apparently solo putative toxins and antitoxin, respectively (Table 1). The reason for the presence of a variety of TA families or the presence of more than one pair of the same TA family per strain is unknown, but there could be crosstalk among them to coordinate the cellular response to various stress conditions [8].

Furthermore, we suggested a putative TA family, GacTA that has not been reported previously in *Geobacillus*. We also identified a putatively new TA composite of the ParDE TA family, AbrB-ParE in three strains. Moreover, our analysis indicated that the XRE-COG2856 TA family, which has not been studied experimentally yet, might be regulated by second messengers, c-di-AMP and (p)ppGpp, and we proposed a hypothesis on the roles of these two messengers in regulating both the gene expression of *xre*-*cog2856* TA pair and the toxin mechanism of action. Additionally, we suggested an approach to abolish the contamination caused by the food spoiling *G. stearothermophilus* in food factories via targeting its TA genes.

Our results indicated that the putative TA families of *Geobacillus* seem to have special characteristics. For example, the putative GacTA TA family has a reverse gene order that has not been reported for the GNAT-HTH TA family, where the toxin gene precedes that of the antitoxin. This gene order renders this putative GacTA family another potentially “unique” TA family. Additionally, we identified putative TA families that seem to feature three components instead of two, in three of the strains. The putative GacTA family of *G. thermodenitrificans* NG80-2 has a putative antitoxin and two adjacent putative toxins, a case that has not been described for the GNAT-HTH TA family. The genes coding for the putative 3-component TA system in this strain seem to be encoded on opposite DNA strands, as explained in the Results and Discussion section. We also found that the genes coding for the XRE-COG2856 family are not fused, which does not seem to be a common pattern for this TA family in prokaryotes [75]. 

All these characteristics make experimental investigation of the type II TA families of *Geobacillus* of significant importance, especially since our knowledge about TAs in thermophilic bacteria in general is very limited.

## Figures and Tables

**Table 1:** TA families, domains, and operons predicted in the current study. Operon prediction was mainly based on the Operon-Mapper tool; exceptions are mentioned. In the last column, the term “Separate” means the toxin and antitoxin are in different operons, while “Shared” means they are located in the same operon. Footnote symbols that are not numbers are consistently used in other tables as well (if present).

Strain	TA Family Name	Antitoxin Domain	Antitoxin Locus Tag	Antitoxin Length (aa)	Toxin Domain	Toxin Locus Tag	Toxin Length (aa)	TA Operon
*Gd* ^†^	GacTA ^1^	wHTH ^¤¤^	GTNG_1350	249	GNAT ^¤^	GTNG_1349	143	Separate ^‡‡^
	GacT, solo toxin	N.A ^±^	N.A ^±^	N.A ^±^	GNAT ^¤^	GTNG_1577	148	Shared with GTNG_1578
	GacT, solo toxin	N.A ^±^	N.A ^±^	N.A ^±^	GNAT ^¤^	GTNG_1578	177	Shared with GTNG_1577
	GacA, solo antitoxin	HTH ^¤^	GTNG_1575	67	N.A ^±^	N.A ^±^	N.A ^±^	Shared with another protein ^‡‡^
	MazEF	RHH ^¤¤^	GTNG_0206	93	PemK/MazF	GTNG_0207	116	Shared
*Gk* ^‡^	GacTA ^1^	wHTH ^¤¤^	GK1499	250	GNAT ^¤^	GK1498	144	Separate ^‡‡^
	MazEF (I)	AbrB/MazE ^¤^	GK1647	89	PemK/MazF ^¤^	GK1648	109	Shared
	MazEF (II)	RHH ^¤¤^	GK0232	93	PemK/MazF ^¤^	GK0233	116	Shared
	ParDE	SpoVT-AbrB (I) ^¤^	GK2355	135	ParE ^¤^	GK2354	111	Shared
	Phd-Doc	SpoVT-AbrB (II) ^¤¤^	GK1845	84	Fic/Doc ^¤^	GK1846	140	Shared
	RelBE	-/XRE ^#^	GK3105	86	RelE ^¤^	GK3104	86	Shared
	VapBC	UPF^2^0175 ^¤¤^	GK1950	96	DUF^3^3368/ COG^4^2405 ^¤^	GK1949	167	Shared
	XRE-COG2856	-/HTH ^ɫ ɫ^	GK3185	130	COG2856 ^¤^	GK3184	264	Shared with 3^rd^ protein ^‡‡^
*Gt* ^§^	GacTA ^1^	wHTH ^¤¤^	*_17290	249	GNAT ^¤^	*_17280	144	Separate ^‡‡^
	MazEF (I)	MazE ^¤^	*_19080	89	PemK/MazF ^¤^	*_19090	109	Shared
	MazEF (II)	RHH ^¤¤^	*_2490	93	PemK/MazF ^¤^	*_2500	116	Shared
	MNT-HEPN (I)	NT^5^/ COG1669 ^¤^ (A)	*_10710	51	DUF^7^86 / COG2361 ^¤^	*_10720	90	Shared
	MNT solo antitoxin	NT^5^/ COG1669 ^¤^ (B)	Unannotated	54	N.A ^±^	N.A ^±^	N.A ^±^	Shared with *_10710 and *_10720
	MNT-HEPN (II)	NT^5^/KNTase ^¤^	*_11510	135	DUF^3^86 ^¤^/ COG2445 ^¤¤¤^	*_11500	139	Shared
	ParDE	SpoVT-AbrB (I) ^¤^	*_26570	122	ParE ^¤^	*_26560	102	Shared ^§§^
	Phd-Doc	SpoVT-AbrB (II) ^¤¤^	*_21520	93	Fic/Doc ^¤^	*_21530	140	Shared operon
	RelBE	-/Xre ^#^	*_34820	86	RelE ^¤^	*_34810	86	Shared^§§^
	VapBC	UPF^2^0175 ^¤^	*_22490	96	DUF^3^3368/ COG2405 ^¤^	*_22480	167	Shared
	XRE-COG2856	-/HTH ^ɫ ɫ^	*_35630	130	COG2856 ^¤^	*_35620	264	Shared with 3^rd^ protein ^‡‡ §§^
ZG ^¶^	GacTA	wHTH ^¤¤^	Contig 16_18	249	GNAT ^¤^	Contig 16_17	144	Separate ^‡‡^
	MazEF (I)	MazE ^¤^	Contig 16_161	89	PemK/MazF ^¤^	Contig 16_162	109	Shared
	MazEF (II)	RHH ^¤¤^	Contig 4_60	93	PemK/MazF ^¤^	Contig 4_61	116	Shared
	MNT-HEPN (I)	NT^5^/ COG1669 ^¤^ (A)	Contig 12_19	51	DUF^3^86 /COG2361 ^¤^	Contig 12_20	90	Shared
	MNT solo antitoxin	NT^5^/ COG1669 ^¤^ (B)	Contig 12_18	54	N.A ^±^	N.A ^±^	N.A ^±^	Shared with 12_19 and 12_20
	MNT-HEPN (II)	NT^5^/KNTase ^¤^	Contig 12_84	135	DUF^3^86 ^¤^/COG2445 ^¤¤¤^	Contig 12_83	139	Shared
	ParDE	SpoVT-AbrB (I) ^¤^	Contig 23_243	135	ParE ^¤^	Contig 23_242	112	Shared
	Phd-Doc	SpoVT-AbrB (II) ^¤¤^	Contig 18_126	93	Fic/Doc ^¤^	Contig 18_127	140	Shared
	RelBE	-/XRE ^#^	Contig 25_196	84	RelE ^¤^	Contig 25_195	86	Shared ^6^

^¤^ The conserved domain was inferred using CDD (Conservation Domain Database) tool; ^¤¤^ The conserved domain was inferred using InterPro domain analysis tool; ^¤¤¤^ COG2445 was inferred using Operon-Mapper tool; * Stands for “GTCCBUS3UF5” that is part of the locus tags in *G. thermoleovorans* CCB_US3_UF5; ^#^ There is no conserved domain in the antitoxin, but it is orthologous to XRE family transcriptional regulator, as shown in the KEGG Genes database/the KEGG KOALA BLAST and explained in the text; ^ɫ^ ^ɫ^ There is no conserved domain in the antitoxin; however, KEGG shows that the protein motif is HTH, as explained in the text; ^‡^^‡^ Details are given in Table S3; ^§^^§^ Operon prediction was based on the ProOpDB. ^†^ *G. thermodenitrificans* NG80-2; ^‡^ *G. kaustophilus* HTA426; ^§^ *G. thermoleovorans* CCB_US3_UF5; ^¶^ *Geobacillus* sp. ZGt-1; ^±^ N.A stands for “not applicable”.^1^ *Geobacillus* acetyltransferase toxin-antitoxin, this TA family name is suggested in this study for *Geobacillus.* strains having HTA-GNAT domain-harboring proteins; ^2^ Uncharacterized Protein Family. ^3^ Domain of Unknown Function, representing protein superfamily; ^4^ Clusters of Orthologous Genes; ^5^ Nucleotidyltransferase domain of DNA polymerase beta-like protein superfamily; ^6^ Operon prediction was inferred manually.

**Table 2:** Previously unrecognized toxins and antitoxins that have been identified in the current study as putatively novel ones. These TAs have either been annotated as hypothetical proteins or have not been annotated.

Strain	Genome Accession Number	Putative T/AT ^1^	Locus Tag	Protein ID ^2^
*Gd* ^†^	NC_009328	RHH	GTNG_0206	WP_008881474 ^3^
*Gk*^‡^ NC_006510		AbrB	GK2355	WP_015375348 ^3^
		HTH	GK3185	WP_011232655 ^3^
		ParE	GK2354	WP_020278248 ^3^
		RHH	GK0232	WP_011229742 ^3^
		XRE	GK3105	WP_011232575 ^3^
*Gt*^§^ NC_016593		AbrB	*_26570	WP_014196297 ^3^
		HTH	*_35630	WP_014196828 ^3^
		MNT solo antitoxin	Unannotated	WP_013146011 ^4^
		ParE	*_26560	WP_014196296 ^3^
		Xre	*_34820	WP_014196753 ^3^
ZG ^¶^ LDPD00000000		AbrB	Contig 23_243	WP_015375348 ^4^
		ParE	Contig 23_242	WP_020278248 ^3^
		RHH	Contig 4_60	WP_011229742 ^3^
		XRE	Contig 25_196	WP_082218538 ^4^

^†^*G. thermodenitrificans* NG80-2; ^‡^ *G. kaustophilus* HTA426. *^§^ G. thermoleovorans* CCB_US3_UF5; ^¶^ *Geobacillus* sp. ZGt-1; * Stands for “GTCCBUS3UF5” that is part of the locus tags in *G. thermoleovorans* CCB_US3_UF5; ^1^Toxin/Antitoxin; ^2^ Represents the RefSeq accession number of the putative toxin/antitoxin protein; ^3^ Accession number belongs to the putative toxin/antitoxin protein as annotated in the RefSeq genome record of the type strain/draft genome sequence of strain ZGt-1; ^4^ Accession number belongs to the NCBI blastp top hit, e-value < 10^−20^.

## Supplementary Materials

Supplementary materials can be found at https://www.mdpi.com/1422-0067/20/23/5869/s1.

## Abbreviations

7TMR-HDED	7 transmembrane helices receptors-HD hydrolase: a hydrolase with a catalytic His-Asp (HD) motif, and ED stands for extracellular domain
aa	amino acid
AbrB domain	AidB regulator domain
c-di-AMP	cyclic-di-adenosine monophosphate
CDD	Conservation Domain Database
Doc	Death on curing
DUF	Domain of Unknown Function
DUF	Domain of Unknown Function
EDF	Extracellular Death Factor
Fic	Filamentation induced by Cyclic AMP
GacTA	*Geobacillus* acetyltransferase Toxin-Antitoxin
GNAT	Gcn5-related N-acetyltransferases
HEPN	Higher Eukaryotes and Prokaryotes Nucleotide-binding
HTH domain	Helix-Turn-Helix domain
KEGG	Kyoto Encyclopedia of Genes and Genomes
KNTase	Kanamycin nucleotidyltransferase
MNT	Minimal Nucleotidyltransferase
nt	nucleotide
NTase	nucleotidyltransferase
PCD	Programmed cell death
Phd	Prevents host death
PIN domain	PilT N-terminus domain
(p)ppGpp	Guanosine tetra or pentaphosphate
ProOpDB	Prokaryotic Operon DataBase
PSK	Post-Segregational Killing
RHH domain	Ribbon-Helix-Helix domain
SpoVT	Stage V sporulation protein T
TA system	Toxin-Antitoxin system
TADB	Toxin-Antitoxin Database
UPF	Uncharacterized Protein Family
VapBC	Virulence associated proteins BC
wHTH domain	winged Helix-Turn-Helix domain
XRE	Xenobiotic Response Element
