# Supplementary material for: Towards Exploring Toxin-Antitoxin Systems in Geobacillus: A Screen for Type II Toxin-Antitoxin System Families in a Thermophilic Genus
Source: Int J Mol Sci. 2019 Nov 22;20(23):5869. doi: 10.3390/ijms20235869 (PMC6929052; doi:10.3390/ijms20235869)
Supplement: Supplementary file 1 [file ijms-20-05869-s001.pdf]

International Journal of *Molecular Sciences*

MDPI

Supplementary material

Towards Exploring Toxin-Antitoxin Systems in *Geobacillus*: A Screen for Type II Toxin-Antitoxin System Families in a Thermophilic Genus

Rawana N. Alkhalili [1,*], Joel Wallenius [2] and Björn Canbäck [2]

[1] Biotechnology, Department of Chemistry, Lund University, SE-221 00 Lund, Sweden;
[2] Department of Biology, Lund University, SE-221 00 Lund, Sweden; J.W. joelwallenius@gmail.com; B.C. Bjorn.Canback@biol.lu.se
* Correspondence: rawana.alkhalili@biotek.lu.se; Tel.: +46 46 222 94 19

Table S1. TAs identified in the current study. The used nucleotide and amino acid sequences belong to the RefSeq genome record of the respective strain, except for GK1498 and GK1499 where the TA pair annotated in the original genome record was used as it showed better alignment.

TA family	Toxin/Antitoxin (T/AT)	Strain	Genome accession #	Locus tag	Protein ID	Protein description
	wHTH_AT	*Gd* [†]	NC_009328	GTNG_1350	WP_008879365	Iclr family transcriptional regulator
	GNAT_T	*Gd* [†]	NC_009328	GTNG_1349	WP_008879366	GNAT family N-acetyltransferase
	GNAT_T [1]	*Gd* [†]	NC_009328	GTNG_1577	WP_008879126	GNAT family N-acetyltransferase
	GNAT_T [1]	*Gd* [†]	NC_009328	GTNG_1578	WP_008879125	GNAT family N-acetyltransferase
GacTA (GNAT-HTH)	HTH_AT [1]	*Gd* [†]	NC_009328	GTNG_1575	WP_008879127	HTH-transcriptional regulator
	wHTH_AT	*Gk* [‡]	BA000043	GK1499	BAD75784	Transcriptional regulator (iclr family)
	GNAT_T	*Gk* [‡]	BA000043	GK1498	WP_011230994 [2]	GNAT family N-acetyltransferase
	wHTH_AT	*Gt* [§]	NC_016593	*_17290	WP_013145506	Iclr family transcriptional regulator
	GNAT_T	*Gt* [§]	NC_016593	*_17280	WP_011230994	GNAT family N-acetyltransferase
	wHTH_AT	ZG [¶]	LDPD00000000	Contig 16_18	Table S2 [3]	Table S2 [3]
	GNAT_T	ZG [¶]	LDPD00000000	Contig 16_17	Table S2 [3]	Table S2 [3]
	MazE (I)_AT	*Gk* [‡]	NC_006510	GK1647	WP_011231142	Abrb/MazE/ SpoVT family DNA-binding domain-containing protein
	MazF (I)_T	*Gk* [‡]	NC_006510	GK1648	WP_011231143	mRNA-degrading endonuclease
MazEF (I)	MazE (I)_AT	*Gt* [§]	NC_016593	*_19080	WP_011231142	AbrB/MazE/ SpoVT family DNA-binding domain-containing protein
	MazF (I)_T	*Gt* [§]	NC_016593	*_19090	WP_011231143	mRNA -degrading endonuclease
	MazE (I)_AT	ZG [¶]	LDPD00000000	Contig 16_161	Table S2 [3]	Table S2 [3]
	MazF (I)_T	ZG [¶]	LDPD00000000	Contig 16_162	Table S2 [3]	Table S2 [3]
	RHH (II)_AT	*Gd* [†]	NC_009328	GTNG_0206	WP_008881474	Hypothetical protein
	MazF (II)_T	*Gd* [†]	NC_009328	GTNG_0207	WP_003253417	Type II toxin-antitoxin system endoribonuclease ndoa
	RHH (II)_AT	*Gk* [‡]	NC_006510	GK0232	WP_011229742	Hypothetical protein
MazEF (II)	MazF (II)_T	*Gk* [‡]	NC_006510	GK0233	WP_003253417	Type II toxin-antitoxin system endoribonuclease ndoa
	RHH (II)_AT	*Gt* [§]	NC_016593	*_2490	WP_011229742	Hypothetical protein
	MazF (II)_T	*Gt* [§]	NC_016593	*_2500	WP_003253417	Type II toxin-antitoxin system endoribonuclease ndoa
	RHH (II)_AT	ZG [¶]	LDPD00000000	Contig 4_60	Table S2 [3]	Table S2 [3]

	MazF (II)_T	ZG [¶]	LDPD00000000	Contig 4_61	Table S2 [3]	Table S2 [3]
MNT-HEPN (I)	MNT (I) A _AT	*Gt* [§]	NC_016593	*_10710	WP_015374218	Putative DNA polymerase beta domain protein region
	HEPN (I)_T	*Gt* [§]	NC_016593	*_10720	WP_014195232	DUF86 domain-containing protein
	MNT (I) B_AT [1]	*Gt* [§]	NC_016593	Unannotated	Table S2 [3]	Table S2 [3]
	MNT (I) A_AT	ZG [¶]	LDPD00000000	Contig 12_19	Table S2 [3]	Table S2 [3]
	MNT (I) B_AT [1]	ZG [¶]	LDPD00000000	Contig 12_18	Table S2 [3]	Table S2 [3]
	HEPN (I)_T	ZG [¶]	LDPD00000000	Contig 12_20	Table S2 [3]	Table S2 [3]
MNT-HEPN (II)	KNTase_AT	*Gt* [§]	NC_016593	*_11510	WP_014195291	Nucleotidyltransferase domain-containing protein
	HEPN (II)_T	*Gt* [§]	NC_016593	*_11500	WP_014195290	DUF86 domain-containing protein
	KNTase_AT	ZG [¶]	LDPD00000000	Contig 12_84	Table S2 [3]	Table S2 [3]
	HEPN (II)_T	ZG [¶]	LDPD00000000	Contig 12_83	Table S2 [3]	Table S2 [3]
ParDE	AbrB (I)_AT	*Gk* [‡]	NC_006510	GK2355	WP_015375348	Hypothetical protein
	ParE_T	*Gk* [‡]	NC_006510	GK2354 [4]	WP_020278248	Hypothetical protein
	AbrB (I)_AT	*Gt* [§]	NC_016593	*_26570	WP_014196297	Hypothetical protein
	ParE_T	*Gt* [§]	NC_016593	*_26560	WP_014196296	Hypothetical protein
	AbrB (I)_AT	ZG [¶]	LDPD00000000	Contig 23_243	Table S2 [3]	Table S2 [3]
	ParE	ZG [¶]	LDPD00000000	Contig 23_242	Table S2 [3]	Table S2 [3]
PhD-Doc	AbrB (II)_AT	*Gk* [‡]	NC_006510	GK1845 [4]	WP_122381439	AbrB/MazE/SpoVT family DNA-binding domain-containing protein
	Doc_T	*Gk* [‡]	NC_006510	GK1846	WP_011231336	Type II toxin-antitoxin system death-on-curing family toxin
	AbrB (II)_AT	*Gt* [§]	NC_016593	*_21520	WP_014195946	AbrB/MazE/ SpoVT family DNA-binding domain-containing protein
	Doc_T	*Gt* [§]	NC_016593	*_21530	WP_014195947	Type II toxin-antitoxin system death-on-curing family toxin
	AbrB (II)_AT	ZG [¶]	LDPD00000000	Contig 18_126	Table S2 [3]	Table S2 [3]
	Doc_T	ZG [¶]	LDPD00000000	Contig 18_127	Table S2 [3]	Table S2 [3]
RelBE	XRE	*Gk* [‡]	NC_006510	GK3105	WP_011232575	Hypothetical protein
	RelE	*Gk* [‡]	NC_006510	GK3104	WP_011232574	Type II toxin-antitoxin system RelE/ParE family toxin
	XRE	*Gt* [§]	NC_016593	*_34820	WP_014196753	Hypothetical protein
	RelE	*Gt* [§]	NC_016593	*_34810	WP_014196752	Type II toxin-antitoxin system RelE/ParE family toxin
	XRE	ZG [¶]	LDPD00000000	Contig 25_196	Table S2 [3]	Table S2 [3]
	RelE	ZG [¶]	LDPD00000000	Contig 25_195	Table S2 [3]	Table S2 [3]
VapBC	UPF0175	*Gk* [‡]	NC_006510	GK1950	WP_013523968	UPF0175 family protein
	COG2405	*Gk* [‡]	NC_006510	GK1949 [4]	WP_021322439	DUF3368 domain-containing protein
	UPF0175	*Gt* [§]	NC_016593	*_22490	WP_013523968	UPF0175 family protein

	COG2405	*Gt* §	NC_016593	*_22480	WP_021322439	DUF3368 domain-containing protein
XRE_COG2856	HTH	*Gk* ‡	NC_006510	GK3185	WP_011232655	Hypothetical protein
	COG2856	*Gk* ‡	NC_006510	GK3184	WP_011232654	ImmA/IrrE family metallo-endopeptidase
	HTH	*Gt* §	NC_016593	*_35630	WP_014196828	Hypothetical protein
	COG2856	*Gt* §	NC_016593	*_35620	WP_014196827	ImmA/IrrE family metallo-endopeptidase

† *G. thermodenitrificans* NG80-2.

‡ *G. kaustophilus* HTA426.

§ *G. thermoleovorans* CCB_US3_UF5.

¶ *Geobacillus* sp. ZGt-1.

* stands for "GTCCBUS3UF5" which is part of the locus tags in *G. thermoleovorans* CCB_US3_UF5.

[1] Solo toxin/antitoxin

[2] The amino acid sequence of the protein product of GK1498 annotated in the original genome record of the strain is 100% identical to WP_011230994 over its entire length.

[3] The nucleotide and amino acid sequences are presented in Table S2.

[4] In the RefSeq genome record of *Gk* strain, only the RefSeq locus tags, GK_RS12120, GK_RS09630, and GK_RS10125 instead of GK2354, GK1845, and GK1949, respectively are shown.

Table S2. Nucleotide and amino acid sequences of the putative TAs of *Geobacillus* sp. ZGt-1 based on the genome sequence annotation of the strain in [26], and of the unannotated putative MNT (II) antitoxin of strain Gts. Genes encoded on the reverse DNA strand have their gene start position value larger than that of the end position. The top hit of blastp results of the TA amino acid sequences is also presented.

**Locus tag**	**nt sequence**	**aa sequence**	**Start position**	**End position**	**Blastp [1]**
Contig 16_18	ATGAATAAAACGGTCTTAAAAACAAAAGAAC<br>TGCTTGATTTGTTTCTCGATTGCGAACGGTTG<br>ACGCTGCCGGAAATGGTCGAGCGGCTTCGGA<br>TGCCCAAAACGTCGGTGTACCGGATGGCGCA<br>GTCGCTTGTTGTCCTTGGTTTTTTGCAAAAAC<br>GAGGCGATTACTATGAGCTCGGTTTAGCCTTC<br>TTGACGTTTGGCGCGCTCGTCGCCGAGCGGC<br>TCGATATTCGCCGGGCGGCGCTGCCGGTGAT<br>GAAGCGGCTGAAAGAGGAGACGAACGAAGC<br>GGTGAATCTTGTCATTCGCGACGGTGATGAG<br>GCGCTGTATATTGAAAAAGTCGAGACGTCCG<br>AGCCGGTGCGCGTCTATACGAAAGTCGGGCG<br>GCGCGCCCCTCTGTATGCCGGGGCGTGCCCT<br>CGTGTCTTGCTCGCGTTTATGGACAAGGCGG<br>ATCGGGAACGTTATTTAGAACAAGTCGAGCT<br>TGTCAAAATCGCCAAACATACGGTGACCGAC<br>AAAGAAGCGCTGCGCCGGTTGTTNGAAGAA<br>GACCGGGACCGCGGCTATACGGTCAGCTATT<br>CTGAGCTGGAAAACTATTCGGCCGCCGTTGC<br>GGTGCCGATTTTCAACCATGAAGGCGCGGCG<br>GTTGCCGGGCTGAGCGTTGCCGGGCCGGAA<br>CAGCGCTTTTCGCCAGATGATGTGGCGCGCA<br>TCGTTCCGCGATTGAAGCAGGCGGCGATGGA<br>CATTTCGCGCGAACTCGGCTTTCGGGGGAAG<br>GGATGA	MNKTVLKTKELLDLFLDCERLTL<br>PEMVERLRMPKTSVYRMAQSL<br>VVLGFLQKRGDYYELGLAFLTFG<br>ALVAERLDIRRAALPVMKRLKEE<br>TNEAVNLVIRDGDEALYIEKVET<br>SEPVRVYTKVGRRAPLYAGACP<br>RVLLAFMDKADRERYLEQVELV<br>KIAKHTVTDKEALRRLXEEDRDR<br>GYTVSYSELENYSAAVAVPIFNH<br>EGAAVAGLSVAGPEQRFSPDD<br>VARIVPRLKQAAMDISRELGFR<br>GKG	17392	18141	WP_047757836; IclR family transcriptional regulator [*Geobacillus* sp. ZGt-1]

Contig 16_17	ATGCCGCAATTTGTTTGGCTCGAGACGGAGGAGGAAGTAAGAAGCGCTTTTCCGGTCATGCGGGAGCTGCGCACTCATTTGGATGAAGAAACGTATGTCGCGCTTGTGCGTGAAGCGCAAGAAAAAGAAGGGTATAAGCTTGCGGCGTTATATGATCAGGACAAAATGGTTGCCGTTGTCGGATTCATGCCGATGATCACGCTCTATAACGGCCGTTTTATTTGGGTTTGCGATTTGGTCACGACATCAGCCGAACGTTCGAAAGGGTATGGAAAAGCGTTGCTGTCGCATGTGCACGAATGGGCGAAGGAGCAAGGCTACGGGATTGTCTCGCTGTCATCCGGCCTGCAGCGGGTTGACGCCCATCGCTTCTATGAGGAAAAAATGGAATATCAAAAAGTGAGTTATGTGTTTTTGAAACGCTTATCGATTTGA	MPQFVWLETEEEVRSAFPVMRELRTHLDEETYVALVREAQEKEGYKLAALYDQDKMVAVVGFMPMITLYNGRFIWVCDLVTTSAERSKGYGKALLSHVHEWAKEQGYGIVSLSSGLQRVDAHRFYEEKMEYQKVSYVFLKRLS	16826	17260	WP_021322570; GNAT family N-acetyltransferase [*Geobacillus*]
Contig 16_161	ATGGAATTAAACAAACAGAAAGGAGTGTCAATCATGACAATAACAGTTCAAAAATGGGGAAACAGCCTTGCGGTTCGCATCCCAAGCGTGATTGCTGAACGTTTAGCGCTTCATCAAGGATCAGAAGTGGAGATGATCGTTGAGAACCAAGCGATCAAGTTGATCCCGAAAAAGAAAAAGCCGACATTGGAGGAACTTTTGGCTAAAATCACGCCGGAAAATCGCCATGCTGAAATTGATTTTGGAACAGAAGGGAATGAATTGTTCTGA	MELNKQKGVSIMTITVQKWGNSLAVRIPSVIAERLALHQGSEVEMIVENQAIKLIPKKKKPTLEELLAKITPENRHAEIDFGTEGNELF	156459	156728	WP_011231142; AbrB/MazE/SpoVT family DNA-binding domain-containing protein [Bacillaceae]
Contig 16_162	ATGCAAGCGCCGGATCGTGGGGATCTTGTTTACGTCAATTTCAATCCACAAGCAGGGCATGAGCAGGCAGGAAAAAGGCCCGGCATCGTTCTATCACCAAAGCGGTTCAATCAATTGACAGGGTTTGCGGTGCTTTGCCCGATCACCCGGCAACAAAAAGGATATCCATTTGAAGTGGAATTGCCATCAGGCTTGGCCGTTGAAGGCGTGATTTTAACCGATCAGGTCAAAAGTTTAGATTGGCGCGCCCGACAGCTTCAAATAGTAGGACGGGCGCCCAATGAAGTTGTTTCGGATTGCTTGGATCTGATTCATACTTTTCTTTCGTGA	MQAPDRGDLVYVNFNPQAGHEQAGKRPGIVLSPKRFNQLTGFAVLCPITRQQKGYPFEVELPSGLAVEGVILTDQVKSLDWRARQLQIVGRAPNEVVSDCLDLIHTFLS	156728	157057	WP_011231143; mRNA-degrading endonuclease [Bacillaceae]
Contig 4_60	GTGTCGGAATCTGGCGCAACAGCGGAAATCGTCGTTCGTTTGCCGCAGTCGCTGCTGACAGAACTGGACGTGCTCGTAAAGCAGGAAAACGGCAACCGCAATGAACTCATTTATCAAGCGACGAAAATGTACATTCGCGAGCGGAAGAAACGGCAAATTCGCGAGGCGATGAGACGAGGCTACATGGAAATGGCGAAAATCAATTTATCTATCGCTTCTGAAGCGTTTCATGCTGAATACGAGGCCGACCACACCGTTGAACGCTTAGTTAGCGGGGGGTAA	MSESGATAEIVVRLPQSLLTELDVLVKQENGNRNELIYQATKMYIRERKKRQIREAMRRGYMEMAKINLSIASEAFHAEYEADHTVERLVSGG	65656	65937	WP_011229742; hypothetical protein [Bacillaceae]
Contig 4_61	TTGATTGTCAAACGTGGCGACGTGTATTTTGCGGACCTTTCCCCGGTTGTTGGCTCGGAGCAGGGCGGCGTGCGCCCCGTGTTGGTGATCCAAAACGATATCGGCAATCGTTTTAGCCCGACGGTGATTGTCGCGGCGATTACGGCGCAAATCCAAAAAGCGAAGCTGCCGACGCATGTCGAGATTGACGCGAAACGCTACGGGTTTGAACGCGATTCGGTCATTTTGCTTGAGCAAATTCGCACGATCGACAAGCAACGGCTGACCGATAAAATCACTCATTTGGACGATGAAATGATGGATAAAGTCGAT	MIVKRGDVYFADLSPVVGSEQGGVRPVLVIQNDIGNRFSPTVIVAAITAQIQKAKLPTHVEIDAKRYGFERDSVILLEQIRTIDKQRLTDKITHLDDEMMDKVDEALQISLGLIDF	65942	66292	WP_003253417; type II toxin-antitoxin system endoribonuclease NdoA [Bacillaceae]

	GAGGCGCTGCAAATTAGCTTAGGGCTGATCG ACTTTTGA				
Contig 12_19	ATGGATAATGCGGTGACGTTTGATCATTATAT GGAGTTGAAACTGAGTTTGGAAGATCATTTT CAAAAACCAGTGGATTTAGTGATTGTAGATG ATATCAAACCAGGATTAAAGCCAACTATTTTA AGGAGTGTTAAGTATGCAGAGGGATCCTAG	MDNAVTFDHYMELKLSLEDHF QKPVDLVIVDDIKPGLKPTILRSV KYAEGS	17787	17942	WP_015374218; putative DNA polymerase beta domain protein region [*Geobacillus*]
Contig 12_18	ATGAAAATAACAGGCGCGGAAAAAGGGGGG GGTTATTTGCTATCACAACAAGACATCCTTAA TGAAATATCGAAATGTTTAGATTTGTGGAAG AAAAAGTATGGTGTGAAGCGAATCGGGTTAT TTGGCTCGTACAGTCGGGGGGGAACAAAGG GAATCAAGTGA	MKITGAEKGGGYLLSQQDILNEI SKCLDLWKKKYGVKRIGLFGSYS RGGTKGIK	17600	17764	WP_013146011; nucleotidyltransferase [*Geobacillus*] [2]
Contig 12_20	ATGCAGAGGGATCCTAGCGTCTTTTTGCAAG ATATTTTGGCTGCTGCAGAGAAAATTGAGAA GTACACCCAAGGGCTTTCCTATGATGACTTTT TAGATAATGATTTAGTATCAGACGCAGTCATT AAAAACATATTAGTGATTGGAGAAGCAGCCA AAAACATTCCAGACGAAATCAGGCAAGCATC CCCATATATCGAATGGAGAAAGATGGCTGGC ATGAGGGATATGCTGATCCACAGTTATTTCTC CATTAATTATCGAATTGTGTAG	MQRDPSVFLQDILAAAEKIEKYT QGLSYDDFLDNDLVSDAVIKNIL VIGEAAKNIPDEIRQASPYIEWR KMAGMRDMLIHSYFSINYRIV	17926	18198	WP_014195232; DUF86 domain-containing protein [Bacillaceae]
Contig 12_84	TTGCCAAACGAAATGGAGACGATCATCATTC AGACGCTCCGCCCGGCTCTTCACCCGTTCGTC ATCTACCTGTTCGGTTCAGCCGCCCGTGGGAC GCTGCGCCCAGACAGCGATGTCGACATCGCC TTTGTCAGCGACGGCGAACCGCATGATCCGT ATGAGCTGTTTCGGCTCGCCGGGGAGTTGGC TGACAAGTTAGGGCGAGATGTCGATCTTGTC GATTTGCGCCAAGCCAGCACCGTGTTTCAAG CGCAAGTCGTCTCGACGGGAAAAGCCATTGA TTGCCGCGACGAGCGGAAACGGGCTGAGTTC GAAATGAAAACATTGAAAATGTATGTGAAAC TAAACGAAGAGAGAGCACCGGTGTTAAAACA AATTACGGAAAGCGGGAGCATATATGAAAA GTGA	MPNEMETIIIQTLRPALHPFVIYL FGSAARGTLRPDSDVDIAFVSD GEPHDPYELFRLAGELADKLGR DVDLVDLRQASTVFQAQVVST GKAIDCRDERKRAEFEMKTLKM YVKLNEERAPVLKQITESGSIYEK	84321	83914	WP_014195291; nucleotidyltransferase domain-containing protein [*Geobacillus*]
Contig 12_83	ATGAAAAGTGATGTCATTTTAAACAAGATCA GCGTGATCGAACGCTGCCTGAAACGAATTCG TGAAGAATATAACGGCGATCCAAAAAATTTA CAAAATTACACAAAACAAGATTCGATCGTTCT GAATTTACAGCGGGCGTGCGAGGCATGCATC GATTTGGCCATGCATATTGTGGCCGAGCAGA AATTCGGATTGCCGCAGCATAGCCGCGATGC ATTCGCTCTCCTCGAAGAACATGGGGTCATCT CCCCTTCCATAAGCAAAAAGATGAAGGCGAT GGTCGGATTCCGCAACATCGCCGTTCACGAC TATCAACAACTGAACCTTGGCATCTTGCAAGC CATCGTCGAACACCATCTTGATGATTTCAAAC AATTTACGAAAGCCATCCTCGATTATGCTAAG AAAAACAGCTAG	MKSDVILNKISVIERCLKRIREEY NGDPKNLQNYTKQDSIVLNLQR ACEACIDLAMHIVAEQKFGLPQ HSRDAFALLEEHGVISPSISKKM KAMVGFRNIAVHDYQQLNLGIL QAIVEHHLDDFKQFTKAILDYAK KNS	83924	83505	WP_014195290; DUF86 domain-containing protein [Bacillaceae]
Contig 23_243	ATGGTACCATCAGATTCGATGACAAAGGAGA AGGTACCGATGGACAATATCCCTAACCGTAA GAAAAACCTTGTGAAACGAATTGCCGTATCG AGCAAAAAACAAATTACGATCCCAAAGGATT	MVPSDSMTKEKVPMDNIPNRK KNLVKRIAVSSKKQITIPKDFYEQ LGIGNEVLIELADNKLIIHPIHED HFDFSDLILKDLIEEGYTGEELYK	238003	237596	WP_015375348; hypothetical protein [*Geobacillus*]

	TTTATGAGCAATTAGGAATTGGAAACGAGGTGTTAATTGAGTTAGCGGATAATAAGTTAATTATTCATCCTATTCATGAGGATCATTTTGACTTTTCCGATCTGATTTTAAAAGACTTGATCGAAGAAGGATATACAGGCGAAGAACTATACAAAGAATTTGTATACCGTAAATCGCAAATTGCCCCTGCATTCAACGCGATGATTTCTGAGGAAAGACCGAAGGCAAAAACGTATACCGCGGATACGTTAGAGGAGTTATTCGGCGAAGATGATGAACAATAA	EFVYRKSQIAPAFNAMISEERPKAKTYTADTLEELFGEDDEQ			
Contig 23_242	ATGATGAACAATAATCGGCTGCAATTGCTCCCAAAAGCAGAAAAAACCATCAAAAAGCTGACAAAGAAAGATCCGGTGCTGAAACAGCGTTTGAAAGAAGCCTTGCGGGAGATTCTTTCCCATCCAACGGAAGCAGGAGAAGCGAAAACAGGGGATTTGGCAGGGATTTACGGTTACGACATTTATCATCAAGGAGTCAATTACGAAATCGCCTATTTTATTGATCAGGATGAAAGCGGGAATATGGTGGTCGTTGTCTTGGCGGGAACCCGCGAAAATTTTTATGATGAGCTGAAGCGTTATATGAAAGCGAACAAAGCGAGACTTCCTAAACCATAG	MMNNNRLQLLPKAEKTIKKLTKKDPVLKQRLKEALREILSHPTEAGEAKTGDLAGIYGYDIYHQGVNYEIAYFIDQDESGNMVVVVLAGTRENFYDELKRYMKANKARLPKP	237609	237271	WP_020278248; hypothetical protein [*Geobacillus*]
Contig 18_126	ATGGACGCGGAAATGTTGGGAAGCAAGGATGACAAAACGTATACACGTCGGATTAGCCAAGTCGGGAATAGTTTGTCCGTTAGCATCCCGAAAGATTTAGCCACGATGCTAAACCTAAATAAAGGCGATGAAATCGAAATATATTACGACAAGGAACGAGGGGAAATCGTGATGAAACGCGCAAACCGAATTCCAAAAGAAGTCCGTCCTGAAGTCGTGATGGCGATGAACCGCGCGATCTCCAAATATGACGAAGCGCTGCGCAACTTGAAATATAGATAA	MDAEMLGSKDDKTYTRRISQVGNSLSVSIPKDLATMLNLNKGDEIEIYYDKERGEIVMKRANRIPKEVRPEVVMAMNRAISKYDEALRNLKYR	124862	125143	WP_013523845; AbrB/MazE/SpoVT family DNA-binding domain-containing protein [*Geobacillus*]
Contig 18_127	ATGGTTTATTATTTGACAGCGGAAGAAATCATATTTATCCATTACACGGTCATGGAAATGTACGACGACGCAGAACAAGCGGGGATCCAATTCCCCGATAAATTCGCGTGGATGTTGGAAAGACCGAAAACGAAATTGTTCGGGGAGGAACAGTTCCCTTCGATTATTGAAAAGGCTTGCTGCTATTACCATTCCATCGCGACAGGTCATATTTTCCACAACGGCAATAAGCGGACGGCTTTAACCGTATTCGTCACGTTCCTTGATTTGAATGGATATGAGTTCACTATGACCAATAAAGAAGCGGAAGATTTCACGGTGTACCTTGTCGAGGATGCCAAGTTCCGGGGAAATGATTGCATCCAGCATCTTGCCCACGAATTAGAGAGTTACATCCGTCCCATCCAAAAACGAAAAGAATGA	MVYYLTAEEIIFIHYTVMEMYDDAEQAGIQFPDKFAWMLERPKTKLFGEEQFPSIIEKACCYYHSIATGHIFHNGNKRTALTVFVTFLDLNGYEFTMTNKEAEDFTVYLVEDAKFRGNDCIQHLAHELESYIRPIQKRKE	125164	125586	WP_014195947; type II toxin-antitoxin system death-on-curing family toxin [Bacillaceae]
Contig 25_196	cntgatatattTTGtAGGAGGacgccntTTGGCTGTGCGCAAACAATTGCTTTACGAACTGATTGAACGGTTGGATGAAACGGATCATCAAACAGCGTACGATTTTTTGATGTACTTGCTTGATCGGTCCAGAAAGGAACGAATGGTATGGGAGCGGATTGACGAAACAGATGAAGAAGAAGCGTTGACGGAAGAAGAGCGCCAGCAGCTGCAAAGCGATGAAGGATATATCACCGGGGGAGAAGCCAAG	MAVRKQLLYELIERLDETDHQTAYDFLMYLLDRSRKERMVWERIDETDEEEALTEEERQQLQSDEGYITGGEAKREFGLQVDLP	191064	190810	WP_082218538; hypothetical protein [*Geobacillus*] [3]

	CGTGAATTCGGGCTACAAGTTGATTTACCGTAG				
Contig 25_195	GTGAATTCGGGCTACAAGTTGATTTACCGTAGGGCCGCAGTCAAATTCATCGCTAGGCAAGAAAAAGAGGTTCAAGAACGGTTGGCCTCTGGGTTGCAAGGTCTGCTTGCGATCCCACCGCAGGGGGATATAAAAAAGTTGAAGGGGCAGGATGGATTATATCGGCTGCGGGTCGGAACATATCGTGTTTTGTTTCGCATCGATCATGATGAACGAATCATCTATATTGAGGCGATCGGCAACCGCGGGGATGTGTATTGA	MNSGYKLIYRRAAVKFIARQEKEVQERLASGLQGLLAIPPQGDIKKLKGQDGLYRLRVGTYRVLFRIDHDERIIYIEAIGNRGDVY	190841	190581	WP_047758268; type II toxin-antitoxin system RelE/ParE family toxin [*Geobacillus*]
Unannotated putative MNT (II); strain Gts [4]	ATGAAAATAACAGGCGCGGAAAAAGGGGGGGGTTATTTGCTATCACAACAAGACATCCTTAATGAAATATCGAAATGTTTAGATTTGTGGAAGAAAAAGTATGGTGTGAAGCGAATCGGGTTATTTGGCTCGTACAGTCGGGGGGGAACAAAGGGAATCAAGTGA	MKITGAEKGGGYLLSQQDILNEISKCLDLWKKKYGVKRIGLFGSYSRGGTKGIK	997713	997877	WP_013146011; nucleotidyltransferase [*Geobacillus*] [2]

[1] Blastp results for all the sequences, except for contig 12_18, and the unannotated putative MNT (II), showed 100% matching identity to the presented protein ID over the entire length and with e-values very close to zero.

[2] The aa sequences of contig 12_18 and the unannotated putative MNT (II) are identical and showed 90% matching identity to WP_013146011, with 94% protein coverage. E-value was very close to zero.

[3] The aa sequence of contig 25_196 showed 99% identity to WP_082218538 over the entire length of the protein. E-value was very close to zero.

[4] The nucleotide and amino acid sequences were predicted by the Operon-Mapper tool. Gene position was retrieved by the tblastn tool.

Table S3. TA operon-sharing genes and their protein products as predicted in this study.

† *G. thermodenitrificans* NG80-2.‡ *G. kaustophilus* HTA426.
§ *G. thermoleovorans* CCB_US3_UF5.

TA family	Strain	Toxin/Antitoxin (T/AT)	Locus tag	Total no. of genes/operon [1]	Locus tag of sharing genes	ID of sharing proteins [2]	Identification of sharing proteins [2]
GacTA	*Gd* †	wHTH_AT	GTNG_1350	5	GTNG_1350	WP_008879364	5-oxoprolinase subunit PxpB
					GTNG_1352	WP_035499192	Biotin-dependent carboxyltransferase
					GTNG_1353	WP_008879362	LamB/YcsF family protein
					GTNG_1354	WP_008879361	Divalent metal cation transporter
	Gd †	GNAT_T	GTNG_1349	1	- [3]	- [3]	- [3]
	Gd †	HTH_AT _solo	GTNG_1575	2	GTNG_1574	WP_008879128	Hypothetical protein
	Gk ‡	wHTH_AT	GK1499	8	GK1500	WP_013145505	5-oxoprolinase subunit PxpB
					GK1501	WP_014195657	biotin-dependent carboxyltransferase
					GK1502	WP_011230998	LamB/YcsF family protein
					GK1503	WP_031206563 [4]	DUF523 domain-containing protein
					GK1504	WP_011231000	Hypothetical protein
					GK1505	WP_011231001	AMP-binding protein
					GK1506	WP_011231002	Phosphotriesterase-related protein
	Gk ‡	GNAT_T	GK1498	1	- [3]	- [3]	- [3]
	Gt §	wHTH_AT	*_17290	5	*_17300	WP_013145505	5-oxoprolinase subunit PxpB;
					*_17310	WP_014195657	Biotin-dependent carboxyltransferase
					*_17320	WP_011230998	LamB/YcsF family protein
					*_17330	WP_015374692 [5]	DUF523 domain-containing protein
	Gt §	GNAT_T	*_17280	1	- [3]	- [3]	- [3]
	ZG ¶	wHTH_AT	Contig 16_18	8	contig16_19	WP_013145505 [5]	5-oxoprolinase subunit PxpB
					contig16_20	WP_014195657 [4]	Biotin-dependent carboxyltransferase
					contig16_21	WP_011230998 [4]	LamB/YcsF family protein
					contig16_22	WP_031206563 [5]	DUF523 domain-containing protei
					contig16_23	WP_011231000 [6]	Hypothetical protein
					contig16_24	WP_011231001 [5]	AMP-binding protein
					contig16_25	WP_021322572 [5]	Phosphotriesterase-related protein
	ZG ¶	GNAT_T	Contig 16_17	1	- [3]	- [3]	- [3]
XRE-COG2856	*Gk* ‡	HTH-COG2856	GK3185 GK3184	3	GK3183	WP_011232653	Hypothetical protein
	Gt §	HTH-COG2856	*_35630 *_35620	3	*_35610	WP_044741960 [4]	Hypothetical protein

¶ *Geobacillus* sp. ZGt-1.

* Stands for “GTCCBUS3UF5” which is part of the locus tags in *G. thermoleovorans* CCB_US3_UF5.

[1] As predicted by the Operon Mapper tool, except for *_35630 and *_35620, whose putative operons were detected using the ProOpDB.

[2] Proteins other than TAs, whose genes are sharing operons with TA-coding genes. The sharing protein IDs and identifications were derived mainly from the NCBI RefSeq genome files with some exceptions which are stated in the next footnotes.

[3] (-) indicates there are no genes, and thus no proteins, sharing the putative operon with the solo T/AT.

[4] Protein ID was derived using blastp; the matching identity was > 99.5% over the entire length, the e-value was equal (or very close) to zero.

[5] Protein ID was derived using blastp; the matching identity was 100% over the entire length, the e-value was equal (or very close) to zero.

[6] Protein ID was derived using blastp; the matching identity was 100% and the protein coverage was 89%, the e-value was very close to zero.

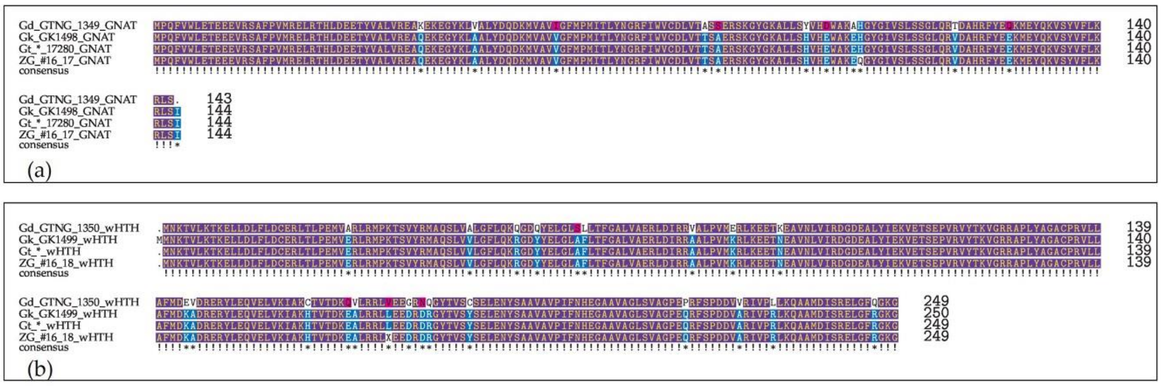

Figure S1. The GacTA TA family. (**a**) Global alignment of the GNAT toxins; (**b**) Global alignment of the wHTH domain-harboring antitoxins.

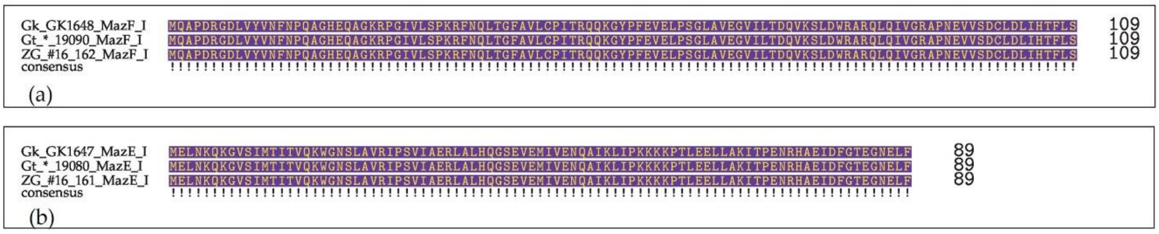

Figure S2. The MazEF (I) TA family. (**a**) Global alignment of the MazF (I) domain-harboring toxins; (**b**) Global alignment of the MazE (I) domain-harboring antitoxins.

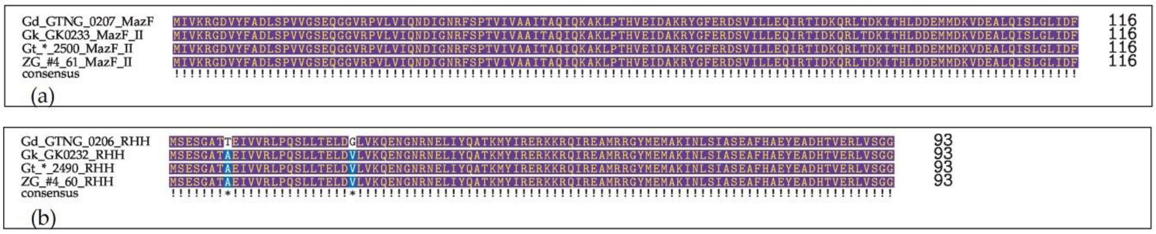

Figure S3. The MazEF (II) TA family. (**a**) Global alignment of the MazF (II) domain-harboring toxins; (**b**) Global alignment of the RHH domain-harboring antitoxins.

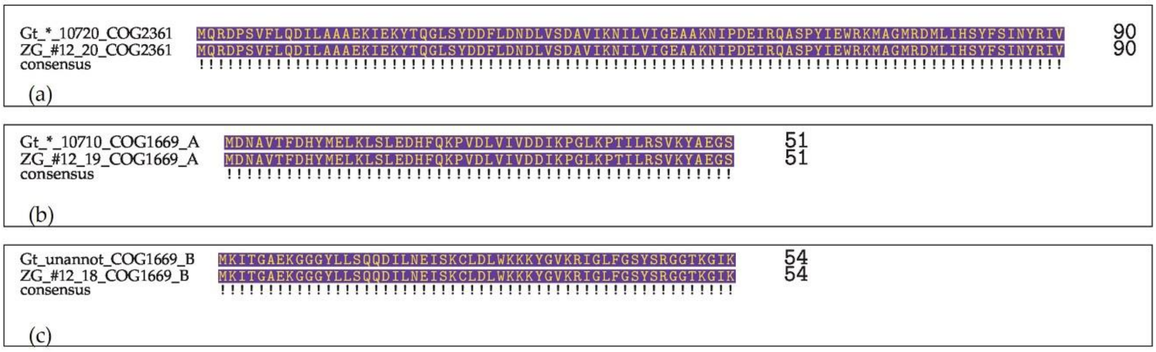

Figure S4. The MNT-HEPN (I) TA family. (**a**) Global alignment of the COG2361 domain-harboring toxins; (**b**) Global alignment of the COG1669 (A) domain-harboring antitoxins; (**c**) Global alignment of the COG1669 (B) domain-harboring antitoxins.

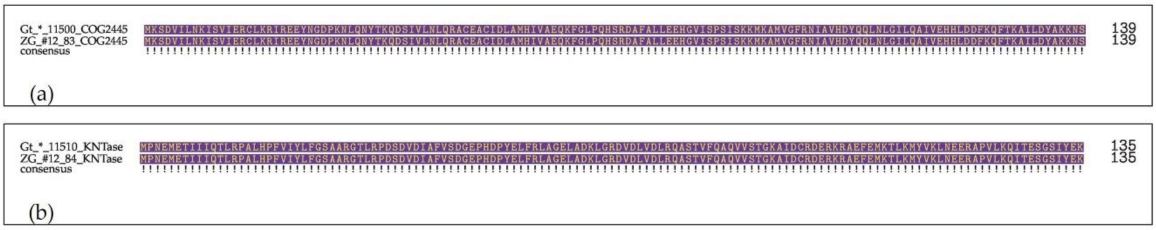

Figure S5. The MNT-HEPN (II) TA family. (**a**) Global alignment of the COG2445 domain-harboring toxins; (**b**) Global alignment of the KNTase domain-harboring antitoxins.

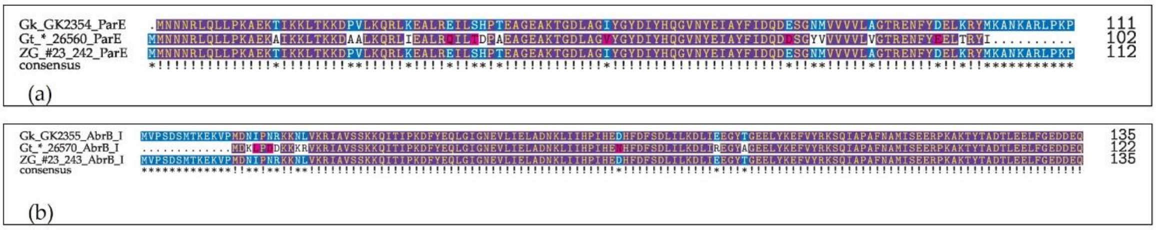

Figure S6. The ParDE TA family. (**a**) Global alignment of the ParE domain-harboring toxins; (**b**) Global alignment of the AbrB domain-harboring antitoxins.

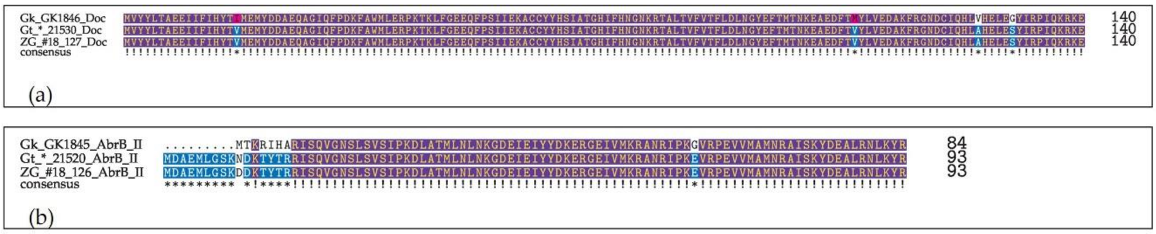

Figure S7. The Phd-Doc TA family. (**a**) Global alignment of the Doc domain-harboring toxins; (**b**) Global alignment of the AbrB domain-harboring antitoxins.

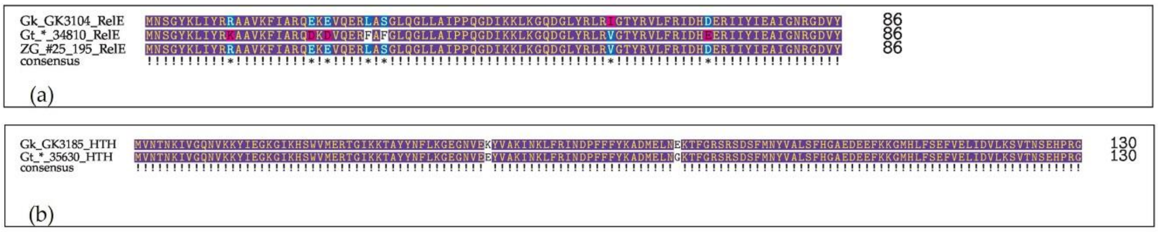

Figure S8. The RelBE TA family. (**a**) Global alignment of the RelE domain-harboring toxins; (**b**) Global alignment of the HTH domain-harboring antitoxins.

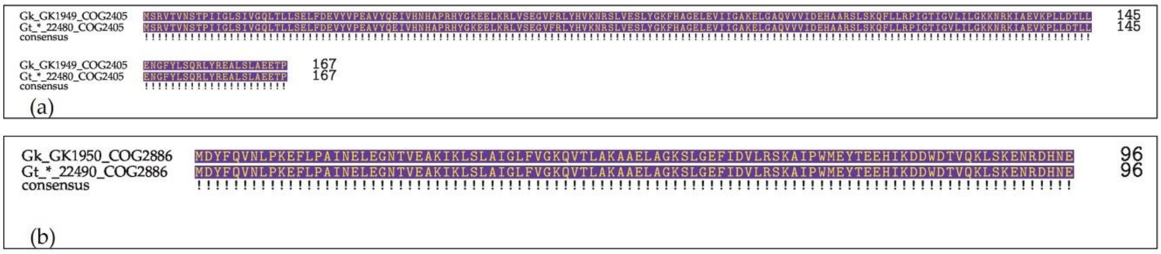

Figure S9. The VapBC TA family. (**a**) Global alignment of the COG2405 domain-harboring toxins; (**b**) Global alignment of the COG2886 domain-harboring antitoxins.

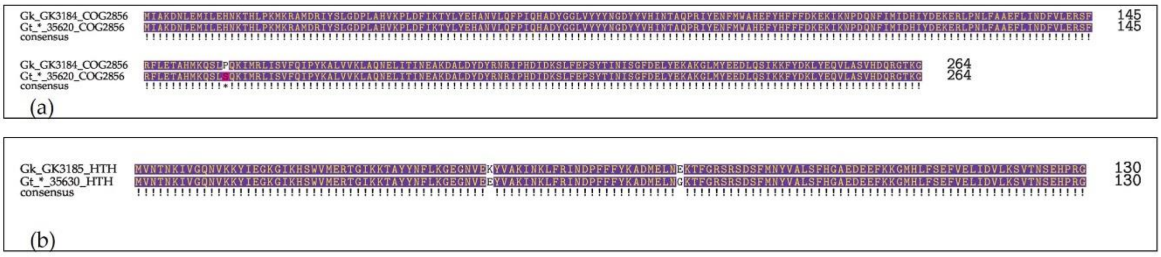

Figure S10. The XRE-COG2856 TA family. (**a**) Global alignment of the COG2856 domain-harboring toxins; (**b**) Global alignment of the HTH domain-harboring antitoxins.

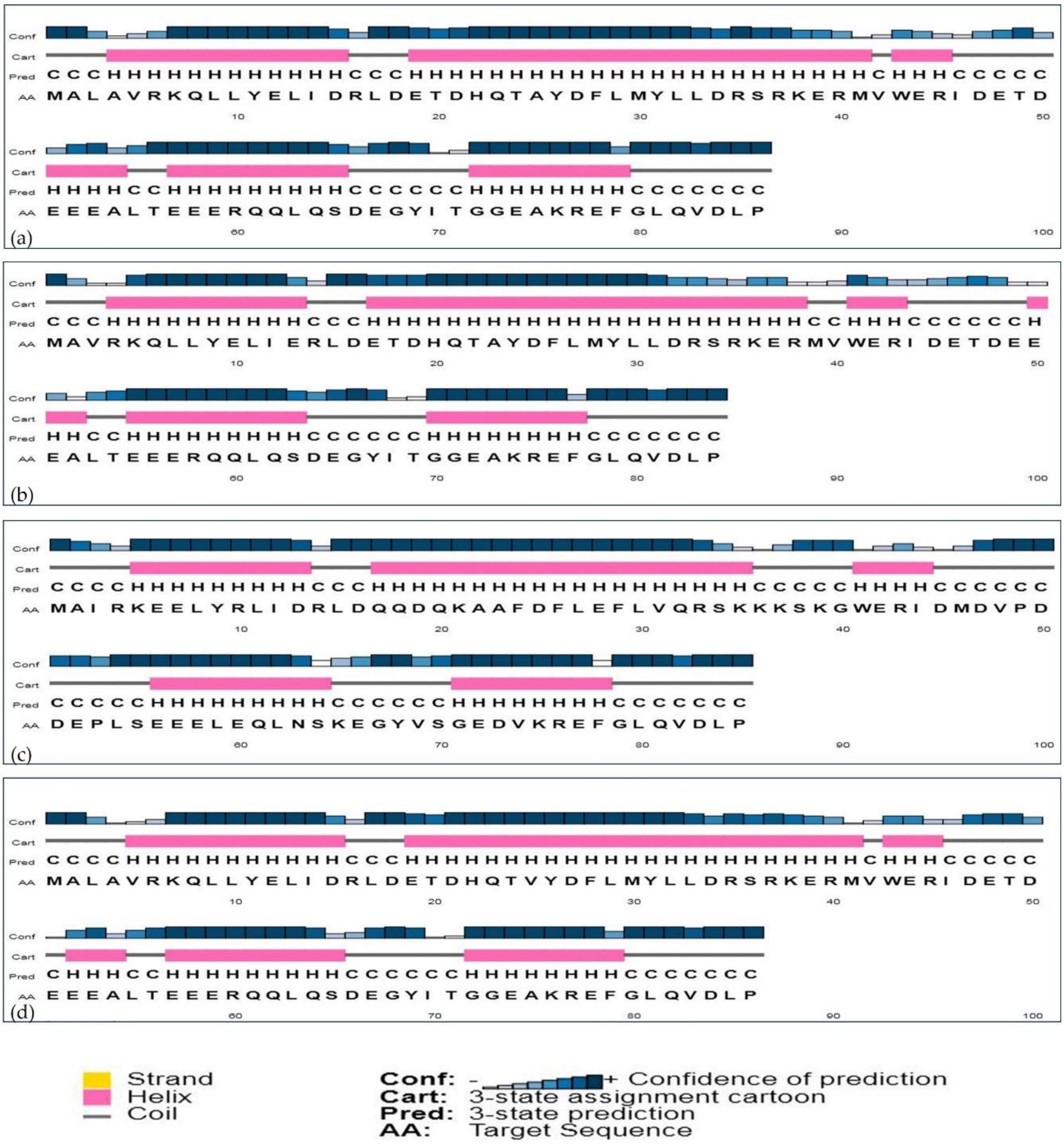

Figure S11. Secondary structure prediction of (**a**) GK3105; (**b**) contig 25_196; (**c**) WP_066367164; (**d**) *_34820 using the PSIPRED workbench tool [145].
